# Engineering the Turnover Stability of Cellobiose Dehydrogenase toward Long-Term Bioelectronic Applications

**DOI:** 10.1021/acssuschemeng.1c01165

**Published:** 2021-05-12

**Authors:** Andreas F. Geiss, Thomas M. B. Reichhart, Barbara Pejker, Esther Plattner, Peter L. Herzog, Christopher Schulz, Roland Ludwig, Alfons K. G. Felice, Dietmar Haltrich

**Affiliations:** † Biocatalysis and Biosensing Laboratory, Department of Food Science and Technology, BOKU − University of Natural Resources and Life Sciences, Muthgasse 18, 1190 Vienna, Austria; ‡ DirectSens Biosensors GmbH, Am Rosenbühel 38, 3400 Klosterneuburg, Austria

**Keywords:** cellobiose dehydrogenase, high-throughput screening, turnover stability, flavoenzymes, direct electron transfer

## Abstract

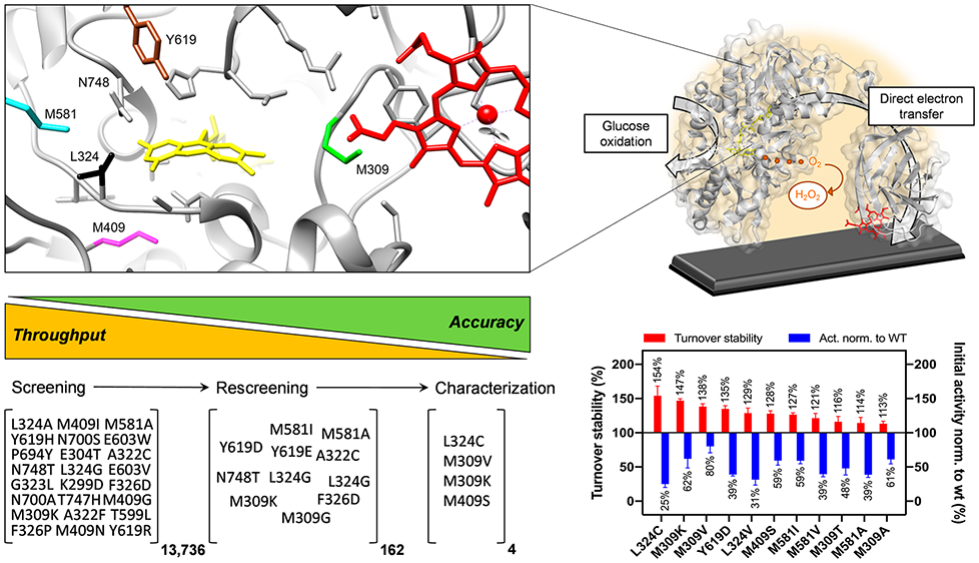

Cellobiose dehydrogenase (CDH) is an attractive oxidoreductase for bioelectrochemical applications. Its two-domain structure allows the flavoheme enzyme to establish direct electron transfer to biosensor and biofuel cell electrodes. Yet, the application of CDH in these devices is impeded by its limited stability under turnover conditions. In this work, we aimed to improve the turnover stability of CDH by semirational, high-throughput enzyme engineering. We screened 13 736 colonies in a 96-well plate setup for improved turnover stability and selected 11 improved variants. Measures were taken to increase the reproducibility and robustness of the screening setup, and the statistical evaluation demonstrates the validity of the procedure. The selected CDH variants were expressed in shaking flasks and characterized in detail by biochemical and electrochemical methods. Two mechanisms contributing to turnover stability were found: (i) replacement of methionine side chains prone to oxidative damage and (ii) the reduction of oxygen reactivity achieved by an improved balance of the individual reaction rates in the two CDH domains. The engineered CDH variants hold promise for the application in continuous biosensors or biofuel cells, while the deduced mechanistic insights serve as a basis for future enzyme engineering approaches addressing the turnover stability of oxidoreductases in general.

## Abstract 2

In this work, we aimed to improve the turnover stability of CDH by semirational, high-throughput enzyme engineering.

## Introduction

Cellobiose dehydrogenase (CDH, EC 1.1.99.18) is an extracellular glycoenzyme of ∼90 kDa secreted by various fungi, both Ascomycota and Basidiomycota.^1^ The enzyme (or more precisely its dehydrogenase domain, DH) is a member of the glucose-methanol-choline (GMC) superfamily of oxidoreductases^2^ and thus shares a common fold with other superfamily members. CDH is a multidomain protein composed of an FAD-containing DH, a cytochrome domain with a heme *b* type cofactor, and in some instances a carbohydrate-binding module. CDH is the only currently known extracellular flavoheme enzyme.^3^ The N-terminal cytochrome domain and the C-terminal DH span about 180 Å in longitudinal dimension^4−6^ and are connected by a ∼20 amino-acid-long linker peptide. This linker is responsible for high domain mobility,^7^ which plays a major role in the function of CDH. CDH oxidizes various sugars, including cellobiose or lactose and in some instances even glucose, at its DH while concurrently electrons are transferred to FAD. Reoxidation of FADH_2_ can occur directly by reduction of various quinones as electron acceptors, or by interdomain electron transfer (IET) to the heme group, from where they can be passed on to cytochrome *c* as an artificial electron acceptor or to lytic polysaccharide monooxygenases (LPMO), the presumed natural interaction partner.^8−10^ Thus, CDH and LPMO are part of an extracellular electron transfer system efficiently fueling the breakdown of recalcitrant lignocellulose by sequential transfer of electrons from soluble sugars via FAD and heme *b* to the active site copper in LPMO.^11^

CDH has attracted significant interest because of its unique electrochemical properties and potential applicability in bioelectronics.^12,13^ CDH is one of the few enzymes that has the structural properties to allow for direct electron transfer,^3,14^ passing electrons directly from its prosthetic heme group to the electrode without the need of a mediator. This can be leveraged to construct a third-generation biosensor architecture, which is characterized by a low working potential effectively omitting many electrochemical interferences observed with other biosensors and resulting in high specificity and robustness.^15,16^ Initially, such sensors were realized on carbon^17,18^ and later also on gold as electrode material.^19,20^ CDH from various fungal organisms can also oxidize glucose efficiently,^21^ which enables the construction of CDH-based biosensors for biomedical applications such as glucose measurements for diabetes management.^15,22^ The initial designs of such biosensors were optimized toward performance under physiological conditions (pH, temperature, etc.) by selection of CDH showing increased glucose activity at neutral pH.^23^ This was fostered by growing insights into the electrochemical properties of CDH from various sources.^24^ Advances in electrode material, e.g., the use of nanostructured gold particles, resulted in improved signal outputs reaching a level where CDH-based sensors or even biofuel cells were successfully employed in complex matrices such as human tears and sweat.^25−27^

Long-term stability of an enzyme is a crucial issue for biomedical or industrial applications since lifetime and cost effectiveness define their commercial viability. CDHs from thermophilic sources show high structural stability; however, this does not guarantee good stability under turnover conditions. CDH stability was found rather moderate when studied in electrochemical setups under turnover conditions, with half-life times in the range of hours up to several days.^14^ Notably, stability in electrochemical applications was strongly improved up to weeks when the enzyme only performed its catalytic reactions repeatedly for short periods of time, such as in a flow injection analysis setup.^28^ In general, three possible modes of destabilization can be conceived for such devices: (i) delamination of the enzyme layer, (ii) deleterious structural reconfiguration of the enzyme layer, or (iii) intrinsic enzyme inactivation.^29^ While structural stability or integrity of enzyme layers can be enhanced by cross-linking or the use of membranes,^30−32^ improving intrinsic enzyme stability, especially under turnover conditions, is more demanding. The enzyme most widely used for bioelectrochemical applications is glucose oxidase (GOX, EC 1.1.3.4). GOX is the gold standard for first-generation glucose measurement systems, which take advantage of the stoichiometric coupling of glucose turnover to the formation of hydrogen peroxide.^33^ However, hydrogen peroxide is a highly reactive oxygen species (ROS), and early studies suggested high sensitivity of the GOX active site, and particularly the transition state, under turnover conditions.^34^ GOX from *Aspergillus niger* was found to show deactivation rates of up to 0.05 h^–1^, which corresponds to a half-life time of activity of only ∼13 h, limiting its use in long-term applications.^35^ More recent studies addressing fundamental inactivation mechanisms of fungal sugar oxidoreductases showed that the loss of activity in the presence of ROS often correlates with irreversible oxidation of certain amino acid residues in the active site.^36,37^ By using mass spectrometry (MS), it was shown that mainly methionine residues are prone to oxidation; however, oxidized forms of histidine and phenylalanine were observed as well.^36,38^

Even though CDH is a dehydrogenase, it shows low activity with oxygen as an electron acceptor (and thus forms H_2_O_2_), and its *k*_cat_ values for oxygen were found to be 2 orders of magnitude lower and *K*_M_ values 4-fold higher when compared to more efficient phenolic electron acceptors.^39^ Nevertheless, in setups where no alternative electron acceptor is available, or sugar substrate is present in excess, H_2_O_2_ accumulation can be significant. Deleterious effects of oxidative damage have therefore also been shown for CDH. Reactor conversion experiments showed a strong dependency of CDH stability on the dissolved oxygen concentration.^40^ Two recent studies described the negative effects of H_2_O_2_ on the stability of CDH, and the authors showed that the replacement of methionine residues can improve the chemical stability of CDH.^41,42^ However, both studies focused on the effects of externally supplied H_2_O_2_, and only limited data are available on the effects of turnover-related ROS production. In general, engineering turnover stability of enzymes is much less explored compared to directed evolution efforts toward higher activities, increased solvent/temperature stabilities, or novel activities.^43−45^

**Figure 1:**
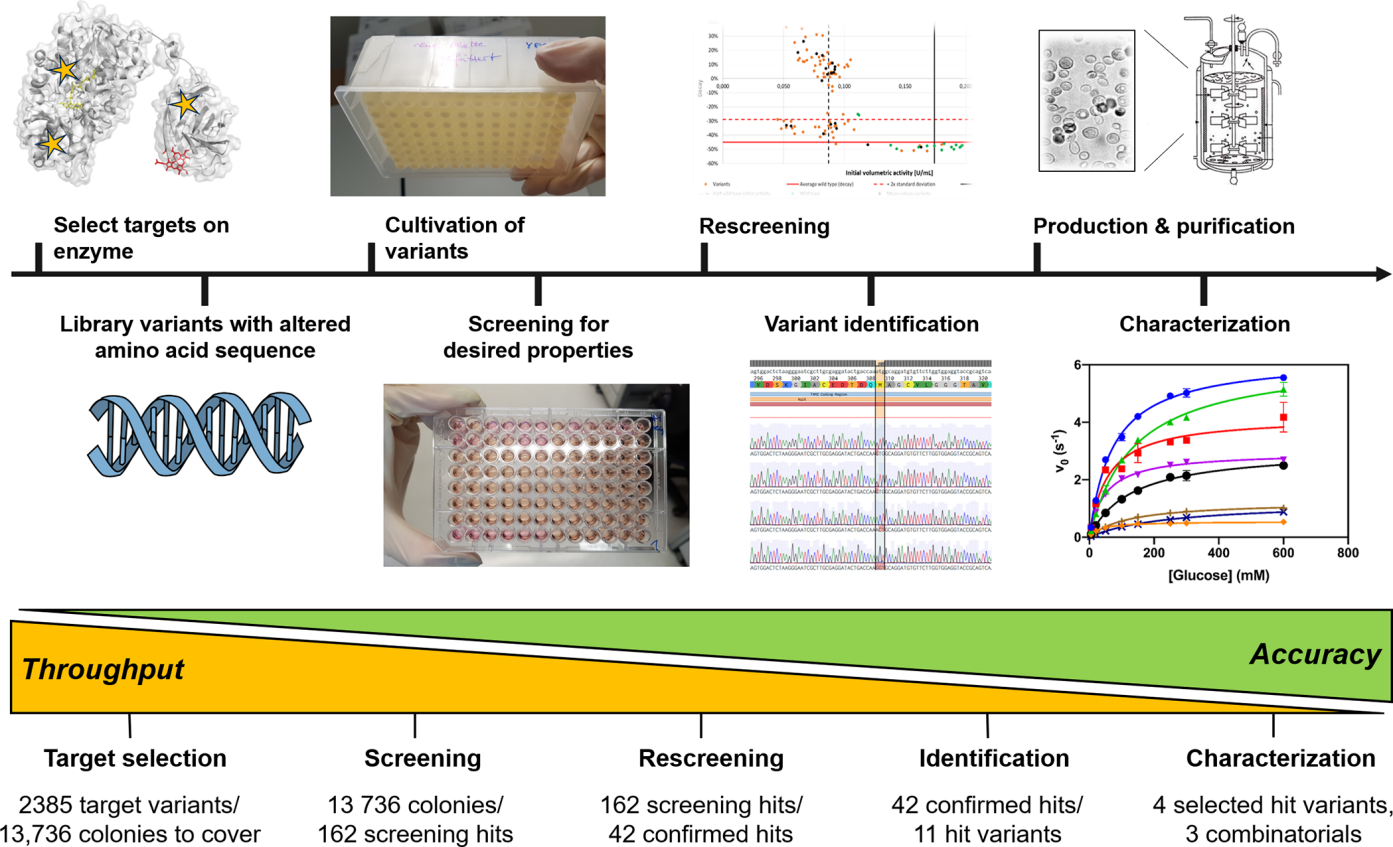
Overview of the process of identifying variants of cellobiose dehydrogenase from *C. hotsonii* with improved turnover stability. The screening process is depicted from the initial target selection of the enzyme for third-generation biosensors to the final biochemical characterization and performance on an electrode.

In this work, we improved the turnover stability of CDH using enzyme engineering based on a semirational design approach combined with high-throughput screening. We hypothesized that CDH turnover stability can be enhanced when (i) amino acid residues affected by ROS are replaced and/or (ii) oxygen turnover by the enzyme is reduced. In this regard, we specifically aimed at improving the kinetic balance of substrate turnover and all subsequent electron transfer steps to prevent a “traffic jam” of electrons at the two cofactors and thus reduce ROS formation. The starting point of the engineering approach was a variant of CDH from *Crassicarpon hotsonii* (formerly *Myriococcum thermophilum*), *Ch*CDH (PDB: 4QI6(7)), which included mutations for improved glucose activity at neutral pH,^46^ subsequently referred to as wild-type CDH (*wt*CDH, WT). Engineering targets were selected by structural comparison with strict sugar dehydrogenases such as pyranose dehydrogenase^47,48^ and homologous residues close to the putative oxygen reaction center in FAD-dependent oxidases.^49−52^ The workflow of the engineering program, ranging from MS-based identification of turnover-mediated oxidation of amino acid residues to library construction, screening, and biochemical as well as electrochemical characterization is illustrated in Figure 1.

## Materials and Methods

### Chemicals

Unless stated otherwise, chemicals were obtained from Sigma-Aldrich or Merck. Solvents and media components were purchased from Carl Roth. Water used was desalted by reverse osmosis (σ = 0.14 μS/cm).

### Molecular Biology

First site-saturation libraries were prepared using NNK primer-based mutagenesis. Subsequently, the small-intelligent library creation method^53^ was applied for obtaining site-saturation libraries with a more homogeneous amino acid distribution. To this end, a set of four mutagenesis primers was used to represent each amino acid by only one codon without stop codons. All primers were designed using the NEBaseChanger online tool. The forward primer was designed to carry the mismatch codon for mutagenesis. Further procedures were done according to the Q5 Site-Directed Mutagenesis Kit (New England Biolabs). The polymerase chain reaction (PCR) program comprised an initial denaturation step at 98 °C for 30 s, followed by 35 cycles of 10 s denaturation at 98 °C, 30 s annealing at the respective annealing temperature given by the NEBaseChanger online tool, and 3 min elongation at 72 °C, terminated by 5 min of final elongation at 72 °C. Template DNA was used at 1 ng together with 10 μM of each forward and reverse primer and the CutSmart buffer provided by the kit at the recommended concentrations. For defined amino acid exchanges, one colony of *Escherichia coli* (*E. coli*) transformants, obtained following the manufacturer’s instructions, was used to inoculate 3 mL of lysogeny broth (LB, 10 g L^–1^ peptone from casein, 5 g L^–1^ yeast extract, 5 g L^–1^ NaCl) liquid culture containing 0.025 mg mL^–1^ Zeocin. The plasmid was harvested after 16–24 h incubation at 37 °C using the Monarch Plasmid Miniprep Kit (New England Biolabs). For site-saturation libraries, all picked colonies were resuspended in 3 mL of LB medium containing 0.025 mg mL^–1^ Zeocin. This resuspension was subjected directly to plasmid preparation following the kit instruction. Plasmids were sequenced to confirm mutagenesis using a commercial sequencing service (Microsynth Austria). Sequences were analyzed using the Benchling online tool (Benchling). Combinatorial libraries of the *Ch*CDH variant gene mentioned above were provided by SESAM Biotech. These included the NAGL loop combinatorial library, shuffled at three positions (A322, G323, L324, each position substituted by A, C, F, G, I, L, M, and V, in a total 511 variants excluding the WT), and the oxygen channel combinatorial library, shuffled at three positions (F326, substituted by C, D, F, H, K, M, N, Q, V, W, and Y; Q597, substituted by C, E, F, H, K, M, N, Q, S, T, W, and Y; T747, substituted by the same amino acids as Q597; in total 1583 variants excluding the WT).

The parent CDH sequence used as a control is consistently referred to as WT regardless of the mutations introduced to improve glucose turnover (C291Y, W295R) and the presence of a C-terminal hepta-histidine tag.

Selected variant-carrying *Komagataella phaffii* (formerly *Pichia pastoris*) cells confirmed by the rescreening were streaked out on yeast extract-peptone-dextrose (YPD, 20 g L^–1^ peptone from casein, 10 g L^–1^ yeast extract, 4 g L^–1^ glucose) agar plates containing 0.1 mg mL^–1^ Zeocin. After incubation at 30 °C for 3 days, cells were transferred into 20 μL of sterile H_2_O using a toothpick, heated to 98 °C for 10 min, then spun down at 16 000*g*. The supernatant (5 μL) was used as template DNA for the following PCR, further comprising 10 μM of forward and reverse primer, each. The colony PCR primers were designed to have annealing temperatures of 72 °C. Apart from that, the same PCR program as described for the mutagenesis was used. The PCR products were purified using the Monarch PCR and DNA Cleanup Kit (New England Biolabs) according to the manufacturer’s instructions. The purified PCR products were sequenced by a commercial provider (Microsynth Austria). Sequences were evaluated using the Benchling online tool (Benchling).

The library plasmid mixtures were transformed into *K. phaffii* cells via electroporation after linearization for 1 h by *Pme*I in CutSmart Buffer (New England Biolabs). To this end, 5 μL of linearized plasmids were added to aliquots of 50 μL electrocompetent ATUM PPS 9011 MutS cells. The device used was the MicroPulser electroporator with Gene Pulser/MicroPulser Electroporation Cuvettes and an electrode gap of 0.1 cm (Bio-Rad Laboratories). The voltage was set to 1.5 kV applied for 3 ms. The transformed cells were resuspended immediately in 500 μL of 1 M ice-cold sorbitol followed by 500 μL of ice-cold YPD. The transformant suspension was incubated at 30 °C for 3 h, then applied to selective YPD agar containing 0.1 mg mL^–1^ Zeocin by using sterile glass beads to obtain single colonies. The agar plates were incubated at 30 °C for 3 days.

### High-Throughput Screening

Autoclaved 96-deep-well plates (MegaBlock 96 well, 2.2 mL, polypropylene; Sarstedt) were filled with 300 μL of YPD medium per well. Single library colonies were used to inoculate each well either automated using the Genetix QPix picking robot (Bioz) or manually using sterile toothpicks. Site-saturation libraries were screened with an oversampling factor of 20 (inoculation of 400 colonies for 20 variants) to ensure the presence of each variant (100% coverage) with a probability of 95%.^54^ The NAGL loop and the oxygen channel combinatorial libraries (511 and 1583 variants excluding the WT, respectively) were screened with oversampling factors of 4 and 3, respectively, according to the manufacturer’s suggestion. Variants containing defined amino-acid exchanges were screened in eight repetitions directly. Each plate contained the control variant (herein referred to as WT, regardless of a 7-histidine tag and the introduced glucose turnover mutations C291Y and W295R) in 16 repetitions and 80 variant colonies per deep-well plate. Master plates were prepared as copy for subsequent rescreening/sequencing. Both screening and master plates were sealed with Breathe-Easy sealing membranes (Diversified Biotech), then cultivated in the HT Multitron incubator (Infors) at 30 °C and 70% humidity. After 3 days, expression was induced by adding 200 μL of a methanol-containing feeding medium (200 mM potassium phosphate buffer, pH 6.0, 3.4 g L^–1^ yeast nitrogen base, 10 g L^–1^ ammonium sulfate, 0.2 mg L^–1^ biotin, 3% (v/v) methanol). After a further 3 days of incubation, the enzyme-containing supernatants were harvested for screening.

The enzyme-containing supernatants were distributed in 40-μL aliquots into two 96-well measuring plates (Greiner Bio-One) using the Biotek Precision pipetting robot (BioSPX) after centrifugation of the deep-well plates (1800*g*, 20 min). The first plate was used to determine the initial CDH activity before the incubation period and the second to determine the residual activity after incubation for 3 h at 37 °C in PBS buffer (11 mM potassium phosphate buffer, pH 7.4, 8 g L^–1^ NaCl, 0.2 g L^–1^ KCl) in the presence of 150 mM glucose.

Screening measurements were conducted in the plate reader Sunrise (Tecan) using the associated data analysis software Magellan, version 5. CDH activity measurements using the cytochrome *c* (cyt *c*) assay (see below) were performed by adding the assay solution (final concentrations of 34.8 mM cyt *c* and 150 mM glucose in 11 mM PBS buffer) to the enzyme samples (final volume of 220 μL), and cyt *c* reduction was followed at 550 nm for 1 min. Volumetric activities were calculated using an extinction coefficient of ε_550 nm_ = 19.6 mM^–1^ cm^–1^.^55^ If the residual activity of a variant surpassed the WT residual activity by more than twice its plate-internal standard deviation, it was defined as a screening hit and subjected to rescreening. Data analysis and visualization were performed in Microsoft Excel and GraphPad Prism, version 8.4.0 for Mac (GraphPad Software LLC). Outliers were removed by the ROUT method.^56^

The rescreening was conducted to confirm that variants selected in the initial screening as hits were not false-positives. The procedure was identical to that described for the initial screening except that each variant was inoculated manually using toothpicks in eight repetitions. The colonies were obtained by single colony separations on YPD agar plates containing 0.1 mg L^–1^ Zeocin from the screening master plates. Confirmed hits were subjected to colony PCR and sequencing as described in the molecular biology section.

### Shaking Flask Cultivation and Enzyme Purification

All CDH variants were recombinantly produced in *K. phaffii* as described previously^57^ in 1 L shaking flasks on 200 mL of YPD medium containing 1.5% (v/v) methanol for induction of the AOX promoter according to the manufacturer’s instructions (Invitrogen). Recombinant protein production was performed for 72 h. Samples were taken daily and tested for enzyme activity and optical density at 600 nm (OD_600_). Supernatants containing the secreted recombinant enzyme were harvested by centrifugation at 17 700 g for 30 min at 4 °C. These crude supernatants were sterile-filtered using 0.22 μm Steritop sterile vacuum bottle-top filters (Merck Millipore). Supernatants were concentrated and rebuffered to 100 mM phosphate buffer, pH 7.4, using a 30 kDa cutoff Hollow Fiber Module (GE Healthcare). His-tagged CDH variants were subsequently purified using immobilized metal affinity chromatography (IMAC) on a prepacked HiTrap IMAC Sepharose 6 Fast Flow column (GE Healthcare) according to the manufacturer’s guidelines. All purification steps were performed on an ÄKTA Pure FPLC system (GE Healthcare). Purified enzymes were concentrated and rebuffered to 1 mM phosphate buffer, pH 7.4, with centrifugal filters (Amicon; 30 kDa mass cutoff) to a concentration of approximately 15 mg mL^–1^ and stored at 4 °C.

### Mass Spectrometry

The proteins were S-alkylated with iodoacetamide and digested in solution with trypsin (Promega). The digested samples were loaded on a BioBasic C18 column (BioBasic-18, 150 × 0.32 mm, 5 μm, Thermo Fisher Scientific) using 80 mM ammonium formate buffer as the aqueous solvent. A gradient from 5% B (80% acetonitrile) to 40% B in 45 min was applied, followed by a 15 min gradient from 40% B to 95% B to facilitate elution of large peptides, at a flow rate of 6 μL min^–1^. Detection was performed with QTOF MS, maXis 4G (Bruker) equipped with the standard electrospray ionization (ESI) source in positive ion/DDA mode (= switching to MS/MS mode for eluting peaks). Mass spectrometry scans were recorded (range 150–2200 Da), and the six highest peaks were selected for fragmentation. Instrument calibration was performed using an ESI calibration mixture (Agilent). The analysis files were converted to mgf files by the device-associated data analysis software, which are suitable for performing an MS/MS ion search with X!-Tandem (Global Proteome Machine Organization). The files were searched against a homemade database. Additionally, manual searches were done and peptides containing methionine were checked for oxidation. Quantification was done by integration of the base peak chromatograms of the monoisotopic peak.

### Enzyme Activity Assays and Protein Quantitation

CDH activity was determined in a plate reader setup by following the reduction of either 120 μM 2,6-dichloroindophenol (DCIP, ε_520 nm_ = 8.97 mM^–1^ cm^–1^, enzyme factor (EF) = 2.14) or 80 μM cyt *c* from equine heart (cyt *c*, ε_550 nm_ = 19.6 mM^–1^ cm^–1^, EF = 0.98) at varying glucose concentrations. In addition, the oxidation of a 50 μM Amplex Red solution (ε_560 nm_ = 54.0 mM^–1^ cm^–1^, EF = 0.356), which is catalyzed by horseradish peroxidase, was followed^55^ as an indirect CDH assay. Enzyme factors (EF) were used as listed to recalculate the raw reading (Abs min^–1^) in a 200 μL scale setup to volumetric activities expressed as U mL^–1^. Samples were buffered with standard PBS buffer and monitored for 300 s on an Infinite plate reader (Tecan) at the respective wavelength and temperatures. The protein concentrations of *wt*CDH and its variants were determined via the absorbance at 280 nm. The theoretical molar absorption coefficient ε_280 nm_ was calculated with Expasy Prot-Param (Swiss Institute of Bioinformatics). Catalytic constants (*K*_M_, *k*_cat_) were derived from nonlinear regression using the Michaelis–Menten equation. Data analysis and visualization were performed in Microsoft Excel and in GraphPad Prism, Version 8.4.0 for Mac.

### Evaluation of Stability

*Storage stability* is defined by the relative residual CDH activity (cyt *c* activity assay) after incubation under defined conditions in the absence of any sugar substrate. *Turnover stability* is defined by the relative residual CDH activity (cyt *c* activity assay) after incubation in the presence of substrate (150 mM glucose) and oxygen/air at given temperatures if not indicated otherwise. *Structural stability* was evaluated by differential scanning calorimetry (DSC) using a MicroCal PEAQ-DSC Automated system (Malvern Panalytical). The thermal transition temperature (*T*_m_) and denaturation onset temperature (*T*_o_) were determined using the MicroCal PEAQ-DSC software. Data analysis and visualization were done using Microsoft Excel and GraphPad Prism, version 8.4.0 for Mac. Outliers were eliminated via the ROUT method.^56^

### Sensor Preparation and Electrochemical Measurements

Selected CDH variants were immobilized on commercial carbon paste electrodes (type DRP-C110; Metrohm/DropSens) following a two-step protocol. First, the electrodes were submersed in a 1% (v/v) solution of ethylene glycol diglycidyl ether (EGDGE; Polysciences) dissolved in 0.1 M NaOH for 1 h at 60 °C. After a washing step with water and drying with nitrogen, 1 μL of enzyme solution (15 mg L^–1^) rebuffered to 1 mM phosphate buffer containing 0.02% Triton X-100 was applied to the working electrode and cured for another hour at 60 °C. Of note, the 60 °C curing step was derived from sensor architecture optimization toward the highest sensor currents (Figure S1). Electrochemical readouts of the modified working electrodes were done using a potentiostat (EmStat3, PalmSens) and a screen-printed carbon counter and Ag|AgCl (0.14 M NaCl) pseudo reference electrode. Current responses to increasing glucose concentrations were measured for sensors vertically immersed in 50 mM potassium phosphate buffer containing 8 g L^–1^ NaCl and 0.2 g L^–1^ KCl, pH 7.4, at 37 °C and at an applied potential of +0.05 V versus the pseudo reference electrode. Current densities were calculated by relating the currents to the working electrode area of 12.57 mm^2^. Square-wave voltammetry was used to derive the midpoint potentials of the CDH cytochrome domain within a potential window of −0.4 to +0.4 V at a frequency of 2 Hz, a step potential of 5 mV, and an amplitude of 30 mV. Cyclic voltammetry was run in the same potential window at a scan rate of 10 mV s^–1^ for two scans.

## Results and Discussion

### Selection of the Mutagenesis Targets

A literature search to identify residues that might modulate the oxygen reactivity in GMC oxidoreductases was conducted. Identified potential mutagenesis targets are summarized in Table 1. In addition to the research *in littera*, we incubated *wt*CDH in the presence of 150 mM glucose and oxygen/air as a sole electron acceptor and followed the oxidation of amino acid residues by mass spectrometry during substrate turnover (Figure S2). The loss of activity over time was determined concomitantly using the cyt *c* assay.

**Table 1:** Rationale for Target Selection

variant	rationale
active site	
K299D	comparison with other CDHs with special regard to charge opposites in the linker region to possibly enhance the interdomain electron transfer
E304S, E304T	comparison with other CDHs with special regard to charge opposites in the linker region to possibly enhance the interdomain electron transfer
E603X	involved in disaccharide binding by a protruding carboxylic group
P694Y	comparison with other CDHs with special regard to charge opposites in the linker region to possibly enhance the interdomain electron transfer
N700X	N700S modulated oxygen activity toward higher peroxide production.^39^ N700 is involved in substrate binding (including monosaccharides) and very close to the FAD isoalloxazine ring. It is well conserved throughout CDHs
putative oxygen reactive center	
oxygen channel combinatorial	putative oxygen channel, predicted by phylogenetic analyses^52^
NAGL loop combinatorial	proximity to N748, which coordinates FAD and modulates oxygen activity^39^
L324X	L324C and L324V were found as screening hits during the NAGL loop screening, which included eight amino acids at this position only; additional variants were checked
F326X	found to be important for oxygen transfer in glucose oxidase; opposite to T750 which complexes FAD^49^
Q597X	glutamine neighboring T599, whose pyranose 2-oxidase equivalent Q448H was found to exhibit reduced oxygen activity^47^
T599X, T599H	pyranose 2-oxidase equivalent Q448H was found to exhibit reduced oxygen activity^47^
Y619X	structural comparison with glucose oxidase and cholesterol oxidase, for which the corresponding position was altered to reduce oxygen activity^49^
T747A, T747H	found as important during previous studies aiming at enhancing oxygen activity of CDH
N748X, N748C	N748 coordinates FAD and modulates oxygen activity;^70^ pyranose 2-oxidase equivalent N593C turned oxidase into dehydrogenase^71^
structural stability	
M75X	M75 complexes the heme cofactor^7^
M309X	mass spectrometry data indicated a high degree of oxidation during substrate turnover within the first hours (Figure S2)
M409X	close to FAD
M513X	oxidation was not observed during substrate turnover by mass spectrometry (Figure S2), hence unclear potential
M581X	in molecular dynamics simulations, oxygen was attracted to a hydrophobic patch of CDH; together with F326 and L324, M581 forms a hydrophobic pocket
M690X, M690L, M690Y	M690L and M690Y were reported as being more stable toward externally added hydrogen peroxide in *Phanarochete crysosporium*, albeit having assessed the DCIP turnover stability only;^58^ showed significant oxidation during turnover in mass spectroscopy (Figure S2)
M703X	proximity to active site

In contrast to previous results, where phenylalanine oxidation was observed,^36^ we only observed the oxidation of methionine residues under these conditions. The *wt*CDH peptide 297–339 showed the most pronounced methionine oxidation within the first 4 h, which points to an effect of the close proximity of this loop to the active site of CDH.^7^ Further, significant oxidation was measured for the peptide fragment 682–694, which contains one methionine (M690), a stability hotspot known for CDH from previous studies.^58^ The authors reported increased stability toward externally added H_2_O_2_ when replacing M690 by either L or Y for the activity assayed with DCIP. However, DCIP is reduced directly at the DH of CDH, and hence its reduction does not involve the interdomain electron transfer, which is essential for the direct electron transfer ability of CDH on electrodes mediated by the cytochrome domain.

Altogether, literature research and mass spectrometric analysis of amino acid oxidation resulted in 2385 target variants to be screened (the “NAGL loop” library of 511 variants, the “oxygen channel” library of 1583 variants, 15 site-saturation libraries of 19 variants, and six single variants; the WT and single variants that were also part of a library were excluded in variant calculations).

### Initial Screening of Mutational Libraries

Growth of *K. phaffii* in 96-deep-well plates (DWP) reached a maximum after 5–7 days with an OD_600_ of ∼25, while the OD_600_ value was ∼5 after 3 days of cultivation (Figure S3). In shaking flask experiments, OD_600_ values reached ∼30 after 3 days. The comparably low growth in DWP was most likely due to limited oxygen transfer. Other difficulties associated with microscale expression and high-throughput screening using *K. phaffii* in DWP include differential growth and variations in gene expression by identical clones in separate wells of the DWP as well as differential stress exposure and varying growth conditions across the plate.^60^ Hence, we applied statistical criteria to improve the reliability of our initial screening process in DWP. We cultivated 16 WT clones on every plate and only rendered the results of that plate valid when 80% of these WT surpassed a threshold of 0.03 U mL^–1^ CDH activity with the cytochrome *c* assay. First of all, this was done as cultivation and expression control. If more than 20% of the WT clones produced activities below 0.03 U mL^–1^, cultivation and expression were considered unsatisfactory. Further, this threshold was applied to WT and variant clones on valid plates to ensure an activity of more than 0.01 U mL^–1^ after the destabilization period, since the assay variance increased significantly at values below 0.01 U mL^–1^ (Figure S4).

**Figure 2:**
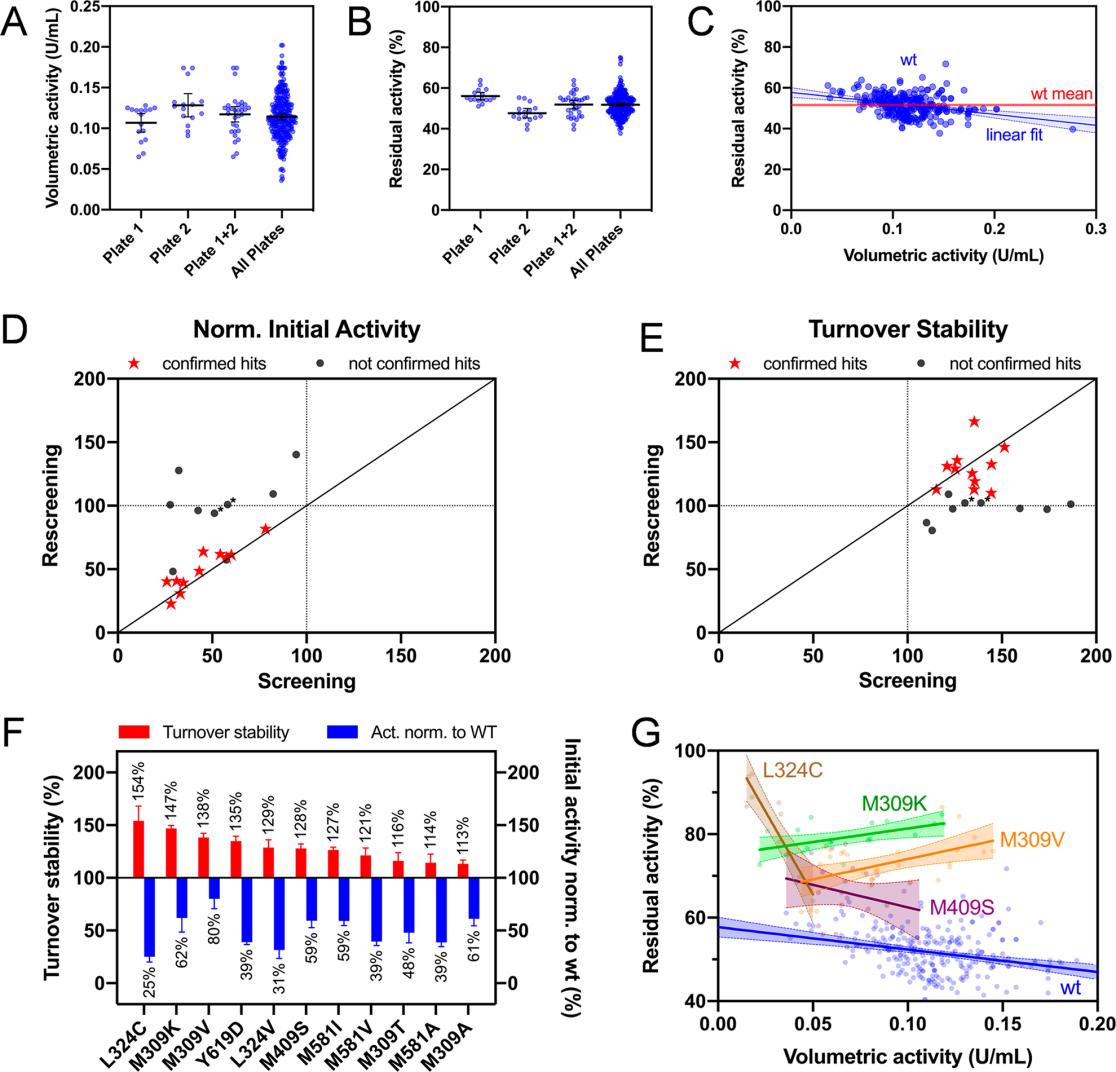
Screening of CDH variants from *C. hotsonii* for improved stability under turnover conditions. (A–C) Screening results: The results for the volumetric activities (A) and residual activities (B) of the wild-type (WT) are analyzed for single plates and the average values measured for two or all plates, respectively. Residual activity is defined as percentage of the initial volumetric activity after a destabilization period of 3 h at 37 °C under turnover conditions in the presence of 150 mM glucose and oxygen/air. Black lines show the arithmetic mean values; error bars give the 95% confidence intervals. Blue dots represent the individual data points. The residual activities of the WT enzyme are related to their respective initial volumetric activities (C). The arithmetic mean of the WT turnover stability is shown as a red line. Linear regression analysis is shown as a blue line with shaded areas depicting the 95% confidence intervals. (D,E) Rescreening results: Confirmed (red stars) and nonconfirmed (gray dots) hits for improved turnover stability (residual activity normalized to the WT) of the screening are compared to the mean values of eight repetitions in the rescreening in terms of their volumetric activity (D) and their turnover stability (E). The asterisks mark WT clones rescreened to validate the rescreening approach. For better clarity, only a few nonconfirmed hits are shown in comparison to the confirmed hits. (F,G) Screening/rescreening results: The volumetric activities and turnover stabilities of the confirmed hit variants are shown as mean values over all measurement data (F). Residual activities of the selected hit variants are plotted against their initial activities (G). Correlation between stability and activity. A linear correlation function is given in the respective variant-assigned color (M309K, green; M309V, orange; L324C, brown; M409S, purple). The WT (blue) linear regression from C is shown for comparison.

Next, we compared the initial volumetric activity obtained for the WT clones in the supernatant before substrate turnover and the residual activity values after 3 h in the presence of 150 mM glucose. These data points were analyzed individually per plate, combined for two randomly chosen plates, and also for the full data set of WT clones from all DWPs. The volumetric activities scattered notably within (*intraplate*) and between the different plates (*interplate*; Figure 2A). To reduce *intraplate* variability, the residual activity after substrate turnover was expressed as a percentage of the initial activity, allowing for improved precision (Figure 2B). Further, we defined a “screening hit” as a variant that surpassed the mean value of the residual activity of the WT controls by at least the 2-fold value of the standard deviation (SD) of the WT controls on the individual plates. This >2 × SD criterion results in a 95% probability that the observed increase in activity is not caused by the variance of the process but by a true improvement. To address *interplate* deviation, the residual activities of screening hits were normalized by the plate-internal WT average. We referred to this computed parameter as *turnover stability* (e.g., Figure 2E,F). Applying these normalizations and criteria, we were able to select and benchmark variants independently of *interplate* and *intraplate* variations.

In Figure 2C, the WT residual activities are plotted against their initial activities to assess whether higher initial activities caused disproportionate activity losses (a higher turnover rate would result in more ROS production). A linear regression analysis was performed to assess a potential correlation between initial activity and residual activity. For the WT, the linear regression deviated significantly from zero (*p* < 0.0001). However, the strength of the correlation (Pearson *r* = −0.32) was very low, indicating a significant yet weak correlation between initial activity and residual activity for the WT. Subsequent monitoring of this effect during screening revealed that for certain variants the apparent turnover stability correlated more strongly (i.e., Pearson *r* < −0.4) with the initial activity (Figure 2G, Figure S5).

A special focus was set on M75, which coordinates the heme *b* cofactor.^7^ Screening the *wt*CDH library M75X resulted in the identification of only a low percentage of active variants (4%) and no stability improvement (Table S1). This was expected (however to be checked), as replacement of the analogous methionine in *Phanerochaete chrysosporium* CDH (*Pc*CDH) by a histidine has been shown to lead to a complete loss of reactivity previously. No activity with one-electron acceptors, such as cyt *c*, which are reduced at the heme domain, was found, while the dehydrogenase function of *Pc*CDH was retained.^59^

### Screening Results Were Confirmed by Rescreening

On the basis of the initial DWP screening, 162 hits were identified that showed improved turnover stability (Table S1). To confirm these hits and exclude false-positives, a rescreening was carried out using the same selection conditions, this time using eight replicates for each clone identified as a screening hit. Exemplary rescreening results of promising variants were compared to their screening results for initial activity (Figure 2D) and turnover stability (Figure 2E). Some of them showed confirmed increased turnover stability compared to the WT in the rescreening (red stars in Figure 2E), with slight variations for individual cases. Others were revealed as false-positives since their rescreening results did not indicate improved turnover stability compared to WT (gray dots in Figure 2D,E). In addition, we performed the rescreening for WT clones that exhibited very high residual activities compared to the average of WT clones (Figure 2B). These were identified as nonconfirmed hit candidates in the rescreening as the residual activity and turnover stability matched that of the average WT (gray dots marked with an asterisk in Figure 2D,E). These results again highlight the necessity and validity of the rescreening approach and underlined the general applicability of our two-step approach.

### Selection for Further Characterization and Combination

After identification by colony PCR, the respective screening and rescreening values could be assigned to the individual variants, enabling statistical evaluation of their performance during the different stages of the process. As shown in Figure 1, 13 736 colonies were screened to cover 2385 target variants. Those 13 736 colonies resulted in 162 screening hits, of which 42 were confirmed. Upon identification of amino acid substitutions via colony PCR, the 42 confirmed hits were reduced to 11 CDH variants, with substitutions at five amino acid positions: M309, L324, M409, M581, and Y619. All hit variants were found at least twice in the screening. The screening hits at the positions M581 and Y619 were not further investigated because of their only moderate stabilization improvement compared to their loss in activity. The averaged performance of the variants ranked by turnover stability is shown in Figure 2F. The entire data set of the hit variants is given in Figures S6 and S7.

Four of the 11 confirmed hit variants were selected for further detailed characterization, as their performance seemed substantially improved compared to the WT. The variants L324C, M309K, M309V, and M409S were selected for medium-scale enzyme production and characterization. Further, hit combinatorials were created to assess potential synergistic effects, M309K/L324C, L324C/M409S, M309K/M409S, and M309K/L324C/M409S. The triple variant was found to be inactive in an expression prescreening (data not shown) and was therefore excluded.

### Confirmation of Screening Results by Purified Enzymes

**Figure 3:**
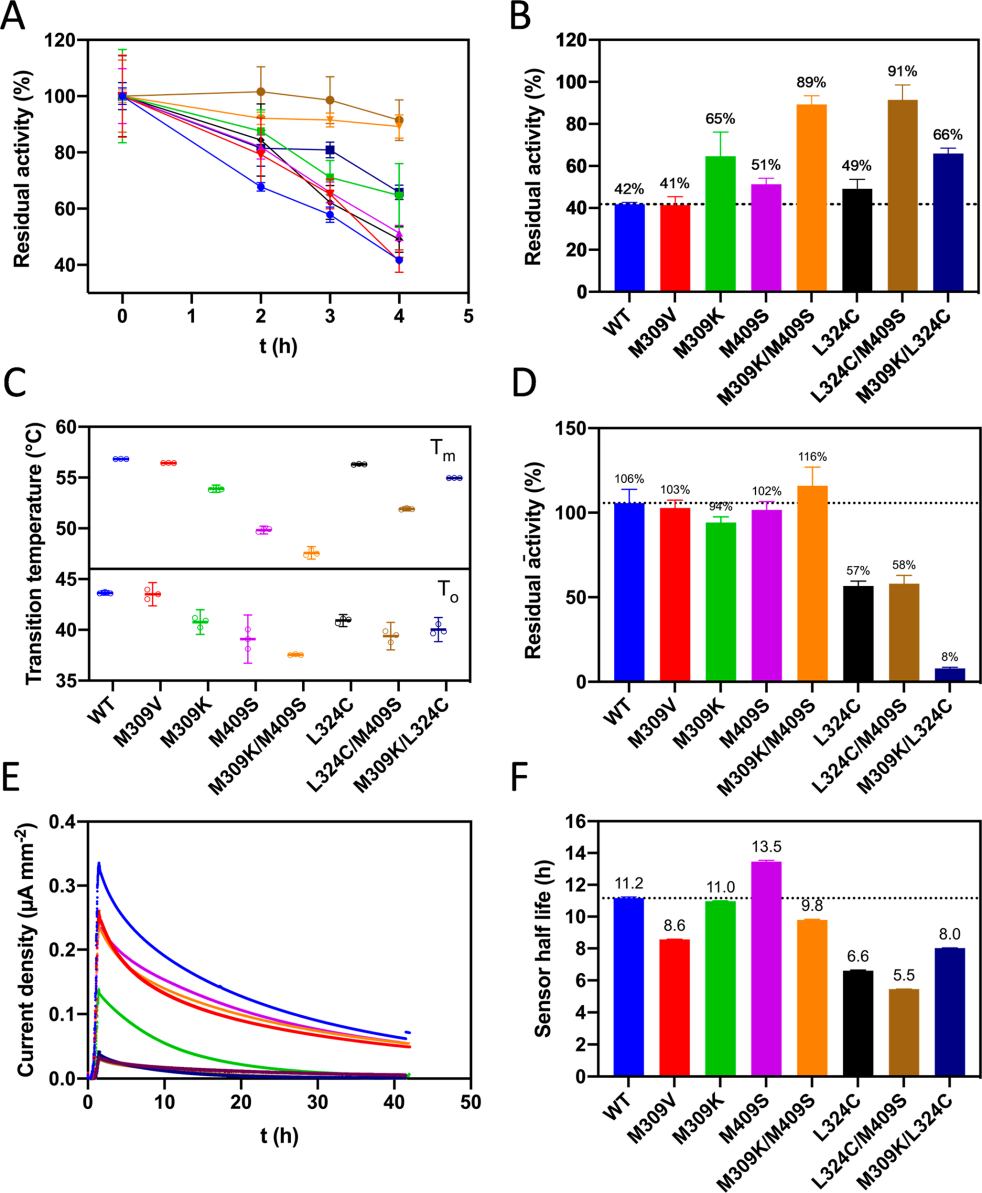
Characterization of improved CDH variants identified during the screening. (A, B) Biochemical turnover stability: Residual activities after inactivation under turnover conditions at 37 °C in the presence of 150 mM glucose (A) and final activity values after 4 h are shown (B). (C, D) Thermodynamic/storage stability: Differential scanning calorimetry (DSC) data are shown with respect to denaturation onset temperature (*T*_o_) and thermal transition temperature (*T*_m_) and compared to the long-term storage stability at 37 °C without glucose after 52 h (D). (E, F) Electrochemical turnover stability: Current densities of CDH-functionalized third-generation electrodes in the presence of 150 mM glucose (E) and their sensor half-life times (F) are shown.

In order to confirm the results obtained in the screening process, which are based on the use of culture supernatants, selected variants were produced in *K. phaffii* cultures using shaking flasks and subsequently purified (Table S2), resulting in apparently homogeneous enzyme preparations as judged by SDS-PAGE (Figure S8). For all variants, the specific activities obtained by the standard cyt *c* assay and glucose as a substrate were reduced when compared to the WT, indicating that the catalytic activity of the variants is affected by the amino acid substitutions. To confirm the screening results with respect to turnover stability, the selected variants (1 mg mL^–1^ in PBS buffer) were subjected to a turnover experiment in conditions similar to the screening in the absence of any electron acceptor but oxygen (Figure 3A,B). Turnover stabilities (Figure S9) and initial activities (Table S3) were in good agreement with the screening data, providing further evidence of the reliability of the screening setup.

### Replacing Methionines Mitigates ROS Effects but Also Reduces Structural Stability

**Figure 4:**
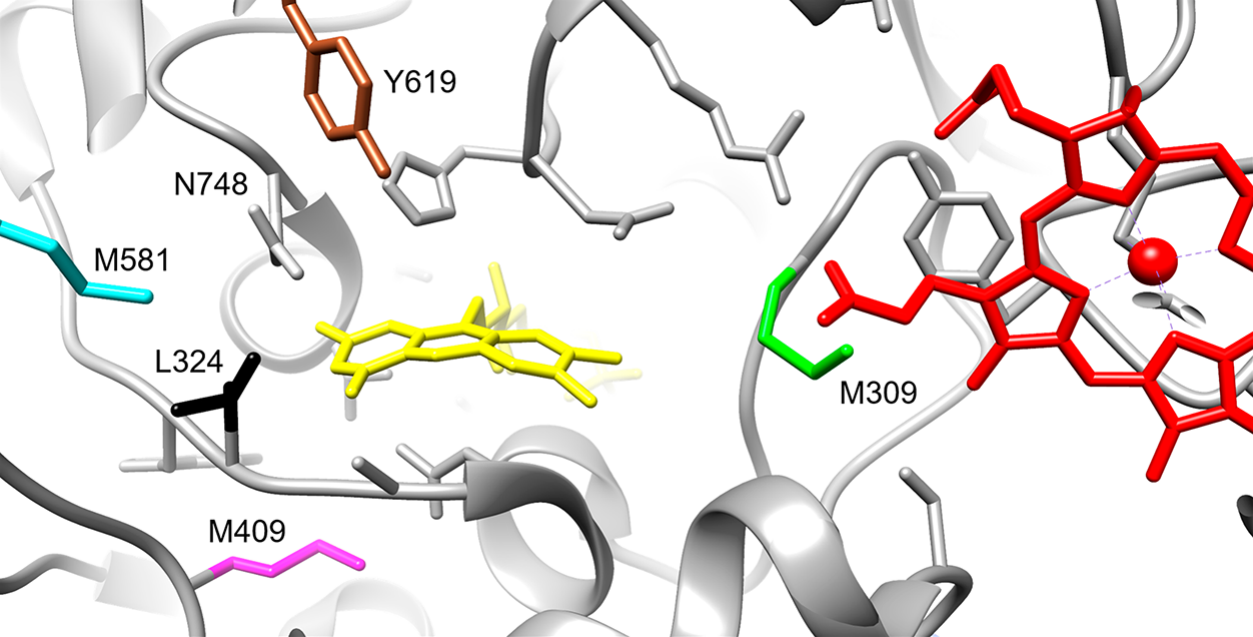
CDH active site. The amino acids, for which substitutions resulted in turnover-stabilized variants (M309, green; L324, black; M409, magenta; M581, cyan; Y619, brown), are shown within the *Ch*CDH crystal structure (PDB: 4QI6(7)). The cofactors FAD and cyt *c* are shown in yellow and red, respectively. The figure was prepared using UCFS Chimera, version 1.13.1.

Improved turnover stability was found for several variants with substituted methionine residues, buried inside the hydrophobic core of the DH domain and near the active site (Figure 4). After the 4 h challenge under turnover conditions, all variants except for M309V showed significant stability improvements compared to the WT, and M309K retained most of its initial activity among the single variants. Significant synergistic effects of substitutions were observed for two of the double variants, M309K/M409S and L324C/M409S, which retained approximately 90% of their initial activity under these conditions (Figure 3A,B). Differential scanning calorimetry (DSC) data (Figure 3C and Figure S10) were evaluated in terms of two properties: the thermal transition midpoint temperature (*T*_m_) and the denaturation onset temperature (*T*_o_). Aiming for *in vivo* applications at 37 °C, both the peak maximum and an increased peak width were considered important since the latter can be indicative for instability caused by higher structural flexibility at lower temperatures. Notably, the thermograms showed two phase transitions. The first phase transition represents the temperature-induced unfolding of the DH domain, which is reduced by the introduction of the two mutations enhancing glucose specificity compared to the native *Ch*CDH.^61^ The second peak depicts the thermal unfolding of the cytochrome domain. Hence, only the *T*_o_ and *T*_m_ of the first transition phase (i.e., unfolding of the DH domain) were considered in our analysis. The WT showed the highest *T*_m_ and *T*_o_ of around 57 and 43 °C, respectively (Figure 3C). All variants but M309V showed significantly lower values, with the double methionine variant M309K/M409S showing the largest effect (*T*_m_ ∼ 47.5 °C, *T*_o_ ∼ 37 °C). Despite these lowered thermal transition temperatures, the CDH variants were found to be more stable than the WT when performing the turnover stability challenge at temperatures of up to 41 °C. Interestingly, M309K/M409S, with a *T*_o_ value of ∼37 °C, showed the highest residual activity after 4 h at 41 °C under turnover conditions (Figure S11), indicating that the positive effect of increased ROS resistance may exceed the beginning effect of denaturation at this temperature. Moreover, the lower catalytic activity of M309K/M409S compared to WT coincides with a reduced rate of ROS formation. No notable effects of the substitutions could be observed when performing the turnover experiment at 45 °C, indicating that the detrimental effects of thermal denaturation exceed the effect of turnover stabilization at this point. Hence, 45 °C could be a general temperature limit for an application of these CDH variants.

Storage stability of CDH variants was determined at 37 °C in PBS in the absence of glucose (Figure 3D and Figure S12). Stability was completely retained after 52 h under these conditions for all variants but those carrying the L324C substitution. L324C and L324C/M409S lost approximately half of their activities within 52 h (57 and 58% remaining activity, respectively), while L324C/M309K was almost completely inactivated (8% remaining activity, Figure 3D). Such discrepancy to DSC data has been observed previously in studies with POX. A high thermodynamic stability was indicated by DSC, but a significant decay was observed in storage experiments at fixed temperature already 10 °C below *T*_o_.^62,63^ We believe this is connected to a minor conformational change in the active site, which cannot be observed in DSC due to the relatively small change in heat capacity compared to the transition phases associated with the protein unfolding process. Retrospectively, L324C could have been excluded earlier in the process as the correlation of increased stability with decreased initial activity was already observed in the rescreening. This correlation can be used as an additional selection criterium in future studies (Figure 2G).

In general, our experiments confirmed that the replacement of methionine residues can improve stability against oxidative damage as shown previously,^42,58^ but it also appears to be coupled to a loss in structural stability not immediately evident at short time scale, especially when these substitutions are within the hydrophobic core of the protein. It has been shown previously that mutations, especially in the core of a protein, frequently result in a decrease of the thermodynamic stability.^64^ This is corroborated by structural studies on *Ch*CDH, which showed that M309 is engaged in several van der Waals interactions with aliphatic amino acid side chains.^7^ Replacing M309 might perturb these interactions, and this seems less pronounced when introducing an aliphatic residue as in M309V (*T*_m_ = 56.4 °C) than for M309K (*T*_m_ = 53.9 °C) with its additional charge. The reduced thermodynamic stability of M409S (*T*_o_ = 39.0 °C and *T*_m_ = 49.8 °C) might be attributed to the destruction of putative methionine-aromatic interactions with three nearby aromatic residues (W325, F408, and F410). The energy associated with such a sulfur–aromatic interaction is comparable to that of a single salt bridge but can occur at longer distances (5–7 Å).^65,66^

### Engineered Variants Show Increased Stability in Electrochemical Setups

To determine the stability in an electrochemical biosensor setup, CDH variants were immobilized on carbon electrodes, which serve as final electron acceptor when polarized sufficiently above the redox potential of the heme *b* cofactor. All variants showed identical redox potentials of −135 ± 5 mV vs Ag|AgCl as determined by square-wave voltammetry, demonstrating that the electric communication between the heme *b* cofactor and the electrode surface is not compromised by the mutations (Figure S13, left column). Catalytic currents induced by glucose conversion were visible for all variants in the cyclic voltammograms (Figure S13, right column) and amperograms (Figure 3E), providing evidence for catalytic, direct electron transfer. In general, current densities compared well to activities in solution, reaching 470 nA mm^–2^ in the case of the WT (Figure S14 and Table S4). After calibration, sensors were run in the presence of 150 mM glucose for 40 h at 37 °C to determine the electrode-associated turnover stability for all variants (Figure 3E). The time course follows an exponential decay, thus rate constants were derived from a fit to a single exponential function and recalculated to half-lives (Figure 3F). In general, the electrode-associated turnover stabilities of all variants were observed to be higher than in the biochemical testing. Biochemical setups differ from electrochemical ones as the electrode serves as an efficient electron acceptor, competing with oxygen as an enzyme substrate. This leads to reduced ROS formation and thus to higher stability. The variant M409S was identified to be ∼20% more stable than the WT in this setup, while other variants, especially the combinatorial variants, suffered higher decays than expected. Thermal stability data described above (Figure 3D) suggest that nonturnover related inactivation processes are predominant over improved turnover stability features over the 40-h measuring procedure of this setup.

### Balance of Kinetic Rates Defines Turnover Stability

**Figure 5:**
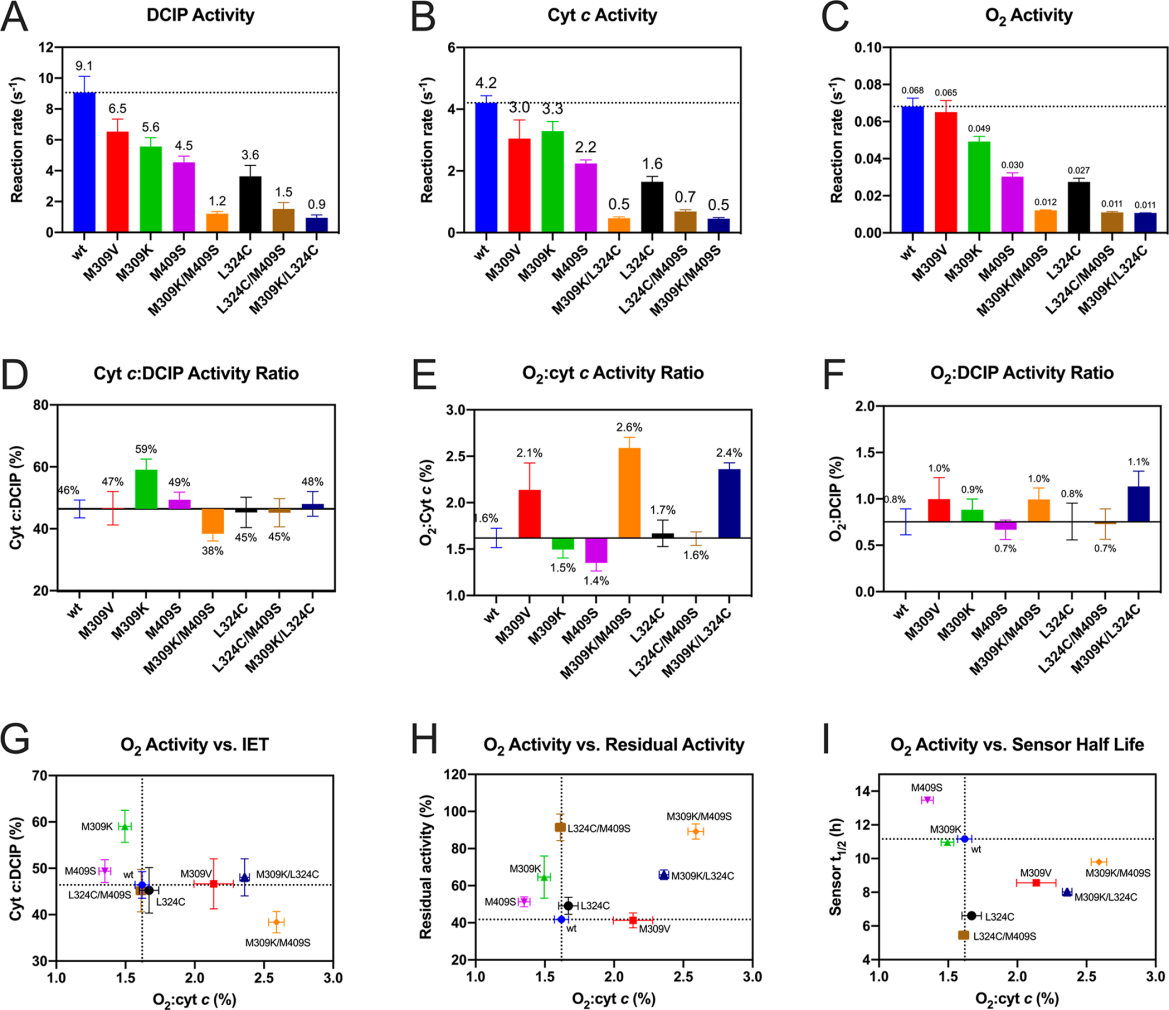
Reaction rates obtained for CDH variants in the presence of 150 mM glucose and using different electron acceptors. (A–C) Reaction rates were determined with the electron acceptors 2,6-dichloroindophenol (DCIP, 120 μM; A), cytochrome (cyt *c*, 80 μM; B), and O_2_/Amplex Red (air saturated O_2_, 50 μM Amplex Red; C) in 11 mM PBS buffer, pH 7.4 at 37 °C with 150 mM glucose. These electron acceptors were used to indicate the activity of only the dehydrogenase domain (DCIP, which is reduced directly at the FAD), the overall activity including the interdomain electron transfer (cyt *c*, reduced at the heme), and the oxygen activity (indirect assay with Amplex Red). (D–F) Activity ratios. The reaction rates at 150 mM glucose are set in relation to each other, cyt *c* to DCIP (D), O_2_/Amplex Red to cyt *c* (E), and O_2_/Amplex Red to DCIP (F). (G–I) Correlations of activity ratios. The reaction rate ratios shown in D–F are set in correlation with respect to kinetic balance between the domains (G), effect of oxygen activity toward biochemical stability (H), and electrode half-life time (I) under turnover conditions.

Last, we determined apparent steady-state kinetic constants for glucose as the varied substrate with three electron acceptors (DCIP, cyt *c*, and O_2_/Amplex Red) in saturating conditions (air saturation for oxygen) to investigate how the mutations affect the individual reaction steps and hydrogen peroxide production (Tables 2, S5 and Figures S15–S17). Cyt *c* is the best predictor for electrode transfer reactions involving the cytochrome domain and is sensitive to perturbations of all previous catalytic steps including the IET from FAD to heme *b*. The WT turnover number with DCIP—exclusively reduced at the DH domain—was higher compared to cyt *c* by a factor of ∼2.5. This was expected, since the IET is known to be rate limiting as resolved in detail by stopped-flow experiments.^67,68^ The Amplex Red assay was used as a measure of oxygen activity, competing with the IET and promoting ROS formation. To correlate the reaction rates to our turnover stability and screening conditions, activities at 150 mM glucose were investigated in detail (Figure 5A–C). All single-point variants showed a reduced reaction rate with both DCIP and cyt *c*, ranging from ∼40% (L324C) to ∼80% (M309V/K) compared to WT (Figure 5A,B). Oxygen was confirmed to be an inefficient electron acceptor, with only 1.7% activity compared to cyt *c* activity in the WT but ranging from 1.0% to 2.9% for the variants (Figure 5C). The combinatorial variants displayed only 10–20% of the WT catalytic activity, suggesting a severe loss of enzyme function but interestingly also showed the lowest oxygen activity (∼15% of WT) compared to the single-point variants (30–50% of WT). To delineate these overlapping effects of decreased catalytic function and oxygen activity, the relative ratios of cyt *c*/DCIP, O_2_/cyt *c*, and O_2_/DCIP activities at 150 mM glucose were calculated (Figure 5D–F). The cyt *c*/DCIP activity ratio was used as a predictor for IET, showing ∼46% for the WT and ranging from 38% (M409K/M409S) to 59% (M309K) for the variants. The O_2_/cyt *c* activity ratio, describing the extent of the oxygen side reactivity, also varied widely (1.4%–2.6%), while the O_2_/DCIP activity ratio is not significantly different for WT and all tested variants. This observation implies that O_2_ activity is primarily influenced by the efficacy of the IET, which stands in competition to the electrons on the FAD cofactor. This effect can be observed to a different extent in each variant.

**Table 2:** Summary of Variant Characterization^a^

property	DCIP		cyt *c*		O_2_(Amplex Red)		activity ratios^b^		DSC^c^		stability		
variant	*k*_cat_	*K*_M_	*k*_c__a__t_	*K*_M_	*k*_c__a__t_	*K*_M_	cyt *c*/DCIP	O_2_/cyt *c*	*T*_o_	*T*_m_	sensor *t*_1/2_	TS^d^	SS^e^
WT	17.4 s^–1^	138 mM	6.3 s^–1^	73 mM	0.105 s^–1^	81 mM	46.4%	1.6%	43.6 °C	56.8 °C	11.2 h	42%	106%
M309V	15.1 s^–1^	196 mM	4.2 s^–1^	56 mM	0.122 s^–1^	130 mM	46.6%	2.1%	43.5 °C	56.4 °C	8.6 h	41%	103%
M309K	13.0 s^–1^	200 mM	6.2 s^–1^	134 mM	0.073 s^–1^	72 mM	59.1%	1.5%	40.8 °C	53.9 °C	11 h	65%	94%
M409S	7.1 s^–1^	84 mM	3.0 s^–1^	48 mM	0.039 s^–1^	41 mM	49.4%	1.4%	39.1 °C	49.8 °C	13.5 h	51%	102%
M309K/M409S	1.6 s^–1^	44 mM	0.5 s^–1^	31 mM	0.013 s^–1^	9 mM	38.4%	2.6%	37.6 °C	47.6 °C	9.8 h	89%	116%
L324C	8.6 s^–1^	205 mM	3.1 s^–1^	130 mM	0.049 s^–1^	118 mM	45.3%	1.7%	40.9 °C	56.3 °C	6.6 h	49%	57%
L324C/M409S	3.7 s^–1^	219 mM	1.2 s^–1^	120 mM	0.012 s^–1^	11 mM	45.2%	1.6%	39.4 °C	51.9 °C	5.5 h	91%	58%
M309K/L324C	3.1 s^–1^	350 mM	1.2 s^–1^	245 mM	0.012 s^–1^	23 mM	48.0%	2.4%	40.0 °C	55.0 °C	8.0 h	66%	8%

a Results of biochemical and electrochemical stability analyses as well as apparent steady-state kinetic constants for glucose in the presence of different electron acceptors at fixed concentrations are shown.

b Activity ratios were calculated using reaction rates measured with 150 mM glucose in 11 mM PBS pH 7.4 buffer in the presence of the stated electron acceptors.

c Differential scanning calorimetry (DSC) was used to determine the denaturation onset temperature (*T*_o_) and the transition phase temperature (*T*_m_). *T*_o_ indicates the onset of denaturation (defined as the temperature where 10% of the enzyme equilibrium is unfolded). *T*_m_ indicates the transition phase temperature at the inflection point of the heat capacity curves where 50% of the enzyme equilibrium is unfolded.

d Turnover stability is defined as the residual activity after 4 h at 37 °C stored in the presence of 150 mM glucose in 11 mM PBS pH 7.4 buffer normalized to the initial activity of the variant.

e Storage stability is defined as the residual activity after 52 h at 37 °C stored in 11 mM PBS pH 7.4 buffer normalized to the initial activity of the variant.

Two variants (M309K and M409S) showed a higher cyt *c*/DCIP activity ratio than the WT, indicating a more efficient IET. At the same time, they also showed a reduced O_2_/cyt *c* activity ratio, indicating improved IET over the reactivity with oxygen (Figure 5G). The effect is stronger for M309K with a higher cyt *c*/DCIP activity ratio (∼59%) than the WT (∼46%) at 150 mM glucose (Figure 5D), which is in agreement with previous stopped-flow spectroscopy showing that M309 mutations influence FAD and heme *b* reduction kinetics.^7^ Notably, the positive charge introduced by the lysine replacement might also contribute to an improved IET as the CDH domain interaction is known to be modulated by surfaces electrostatics.^61,69^

M409S is the only variant showing a higher sensor stability than the WT (Figure 3F). This was surprising as M409S was the screening hit with the lowest turnover stability among all the selected variants (Figure 2F) and also showed the lowest thermodynamic stability among all single-point variants in DSC (Figure 3C). However, M409S has the lowest O_2_ activity (Figure 5C) among the single-point variants and smallest O_2_/cyt *c* ratio of all variants (Figure 5E). Furthermore, it is the methionine residue closest to the isoalloxazine moiety of FAD with an S-to-N5 distance ∼9.4 Å that might be affected by ROS. Combining M309K and M409S reduced O_2_ activity even further (Figure 5C) and improved its turnover stability in solution (Figure 3B) but led to a near complete loss of catalytic activity (Figure 5A,B) together with a decrease in thermodynamic stability (Figure 3C) and electrochemical performance (Figure 3F).

On the basis of these findings, we propose two mechanisms, by which M309K and M409S improve the turnover stability of CDH. First, the removal of M309 or M409, which can be oxidized by ROS, increases the oxidative stability of CDH in solution (Figure 3A,B). Second, the mutations reduce O_2_ reactivity compared to the overall activity, thus lowering the formation of detrimental ROS under turnover conditions (Figure 5E), especially when IET is competing with electron transfer to oxygen. Both mechanisms are supported by M309V showing a similar turnover stability as the WT (Figure 3B) even though the O_2_/cyt *c* ratio is increased (Figure 5E and H). This indicates that the sole removal of the ROS-sensitive target M309 improves the stability against ROS damage. Moreover, reduced oxygen reactivity correlates well with the electrode half-life times (Figure 5I). Variants showing lower sensor half-lives revealed higher O_2_/cyt *c* activity ratios, with variants carrying the false-positive hit L324C being a notable exception for reasons described above. M409S, the most stable variant with an electrode half-life of 13.5 h, also showed the lowest O_2_/cyt *c* activity ratio (1.4%). These findings support our hypothesis that removing ROS targets and reducing oxygen reactivity are effective strategies to improve the sensor lifetime. Overall, the improved catalytic balance of M309K and M409S suggests that amelioration of an “electron traffic jam” in the DH might be a crucial mechanism to prevent the FAD cofactor being in the reactive semiquinone form, by which turnover stability of CDH can be improved.

## Summary and Conclusion

We introduced a screening setup that allows for variant selection of the oxidoreductase CDH in terms of turnover stability. A total of 2385 target variants were designed based on rationales from previous studies and mass spectrometry results. A total of 13 736 colonies were screened to cover all target variants and subsequently narrowed down to 162 screening hits, 42 confirmed hits, and finally 11 variants at five distinct positions. The screening process was confirmed by experiments with purified enzymes tested at fixed concentrations in defined buffers. We further determined biochemical as well as electrochemical stability, and derived apparent Michaelis–Menten kinetic constants for glucose with three different electron acceptors to elucidate the destabilizing mechanism. In the electrochemical long-term measurement setup, one variant (M409S) was confirmed to be 20% more stable compared to WT. This position was predicted by mass spectrometry to be prone to oxidation, and we conclude that methionine oxidation close to the active site strongly contributes to long-term turnover stability in oxidoreductases. All other variants were notably turnover-stabilized in biochemical characterization but less stable on the electrode and in thermodynamic assays. Thus, replacements of methionines can enhance turnover stability but often lead to structural destabilization of the native conformation, which becomes evident at extended time scales or increased temperature. In general, we found higher stability in electrochemical setups, where the electrode serves as a constant, efficient electron acceptor, repressing the oxygen side reactivity and ROS formation in CDH. We initially hypothesized that an improved balance of the catalytic reactions at the two domains and thus more efficient IET can further improve the stability, which is supported by an extensive kinetic analysis of our results.

In summary, the presented screening resulted in one CDH variant that exhibited improved turnover stability on a biosensor electrode, which is suitable for the application in implantable continuous glucose monitoring biosensors or biofuel cells. The deduced mechanistic insights serve as an excellent basis for future enzyme engineering approaches, addressing the turnover stability of oxidoreductases.

## Supporting Information Available

Mass spectrometry, assay and screening validation, screening results, protein production, differential scanning calorimetry, biochemical stability assays, electrochemical characterization, and Michaelis–Menten kinetics (PDF)

## Abbreviations

DCIP: 2,6-dichloroindophenol
DWP: 96-deep-well plate
CDH: cellobiose dehydrogenase
*Ch*CDH: *Crassicarpon hotsonii* CDH
cyt *c*: cytochrome *c*
DH: dehydrogenase domain
DSC: differential scanning calorimetry
EGDGE: ethylene glycol diglycidyl ether
FAD: flavin adenine dinucleotide
GOX: glucose oxidase
IET: interdomain electron transfer
LB medium: lysogeny broth medium
LPMO: lytic polysaccharide monooxygenase
MS: mass spectrometry
PBS buffer: phosphate-buffered saline
*Pc*CDH: *Phanerochaete chrysosporium*
POX: pyranose 2-oxidase
ROS: reactive oxygen species
WT: wild-type
YPD medium: yeast extract-peptone-dextrose medium
