# Supplementary material for: Engineering the Turnover Stability of Cellobiose Dehydrogenase toward Long-Term Bioelectronic Applications
Source: ACS Sustain Chem Eng. 2021 May 12;9(20):7086–100. doi: 10.1021/acssuschemeng.1c01165 (PMC8296668; doi:10.1021/acssuschemeng.1c01165)
Supplement: Supplementary file 1 — sc1c01165_si_001.pdf [file sc1c01165_si_001.pdf]

Supplementary Information

Engineering the turnover stability of cellobiose dehydrogenase towards long-term bioelectronic applications

Andreas F. Geiss[1‡], Thomas M.B. Reichhart[1,2‡], Barbara Pejker[1], Esther Plattner[2], Peter L. Herzog[2], Christopher Schulz[2], Roland Ludwig[1,2], Alfons K.G. Felice[2] and Dietmar Haltrich[1]*

[1] Biocatalysis and Biosensing Laboratory, Department of Food Science and Technology, BOKU – University of Natural Resources and Life Sciences, Muthgasse 18, 1190 Vienna, Austria

[2] DirectSens Biosensors GmbH, Am Rosenbühel 38, 3400 Klosterneuburg, Austria

* To whom correspondence should be addressed: Alfons K.G. Felice, DirectSens Biosensors GmbH, Am Rosenbühel 38, 3400 Klosterneuburg, Austria; E-mail: alfons.felice@directsens.com; Telephone: +436505000167

‡These authors contributed equally

Number of Pages:19 Number of Figures:17

Number of Tables: 5

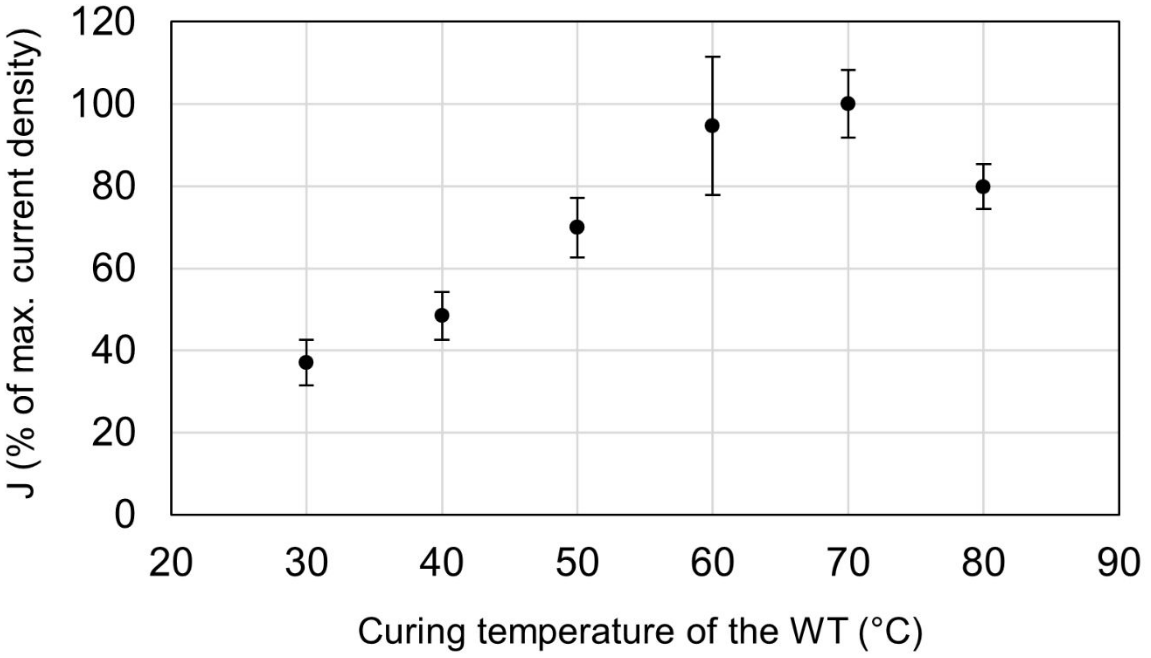

Figure S1: Dependence of catalytic current densities (J) for 20 mM glucose at 0 V vs. Ag|AgCl in PBS at 37 °C on the curing temperature of the wtCDH added to the EGDGE activated electrodes.

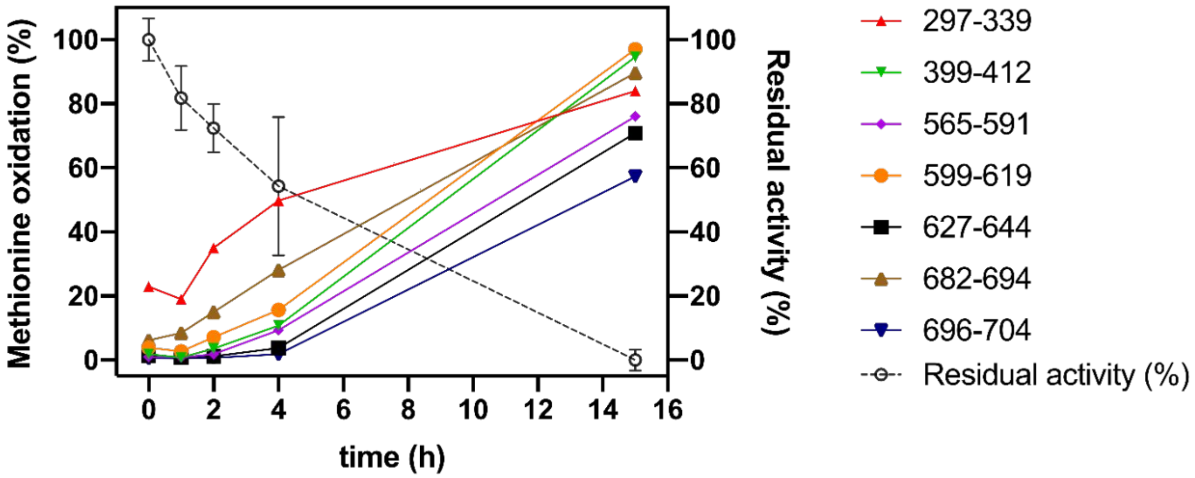

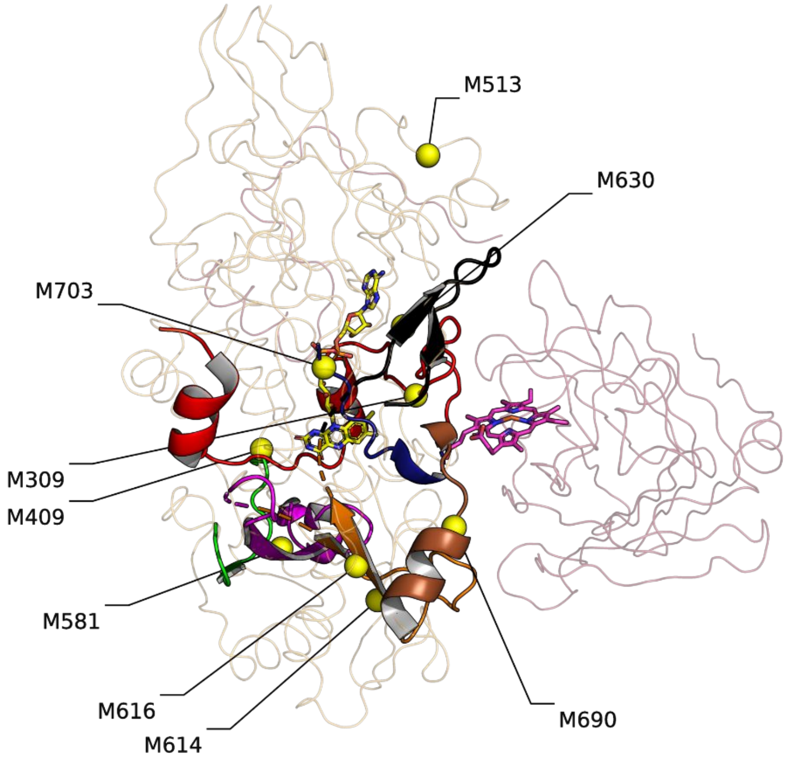

Figure S2: Mass spectrometric analysis of methionine oxidation in the dehydrogenase domain of cellobiose dehydrogenase. Top: Fraction of oxidized methionine residues in *Ch*CDH samples incubated in 11 mM PBS buffer at pH 7.4 and 37 °C in the presence of 150 mM glucose for 1, 2, 4, and 15 h. Methionine oxidation was measured by mass spectrometry. The residual enzymatic activity was determined via the cytochrome *c* assay over the same time frame. The only methionine residue that could not be identified by mass spectrometry is M513, which is located within the peptide segment 506-557. Bottom: Fragment locations in the CDH crystal structure

(PDB: 4QI6 (Tan et al., 2015)) using the same color code as in the top graph. The protein backbone is shown as ribbons and the fragments analyzed in MS are shown as cartoons. The two prosthetic groups FAD and heme *b* are represented as yellow and magenta sticks, respectively. Alpha carbon atoms of methionine residues on the DH domain are shown as yellow spheres. Images were prepared with the PyMOL Molecular Graphics System (version 2.3.4, Schrödinger, LLC).

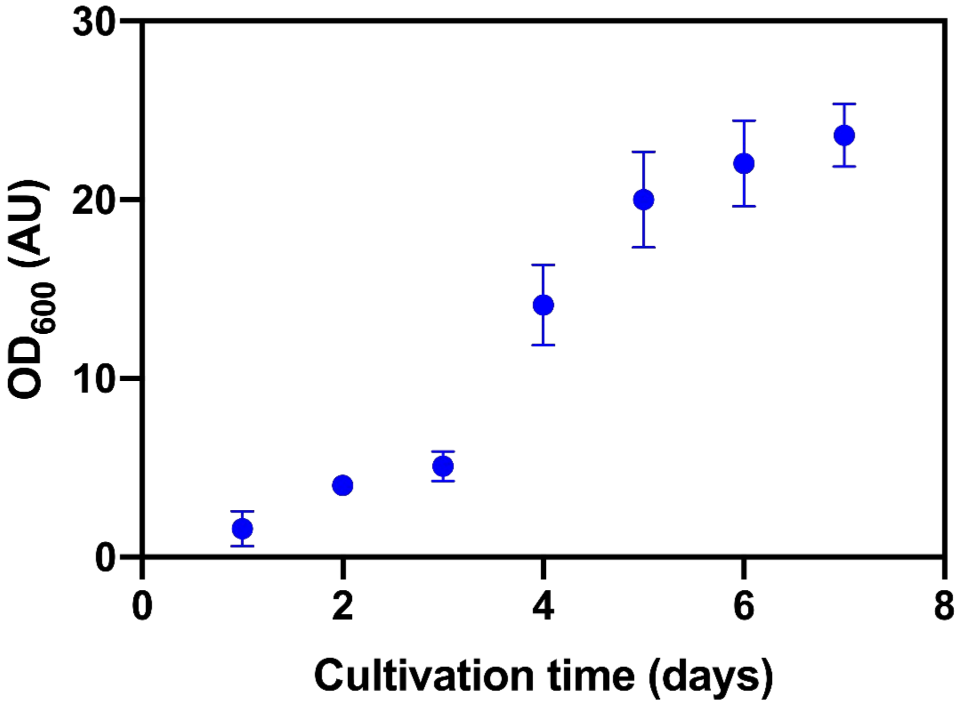

Figure S3: Cell densities obtained for *K. phaffii* 96-deep-well cultures. The content of three wells was harvested per day to determine the OD_{600} values. The mean and standard deviation of triplicate measurements are shown.

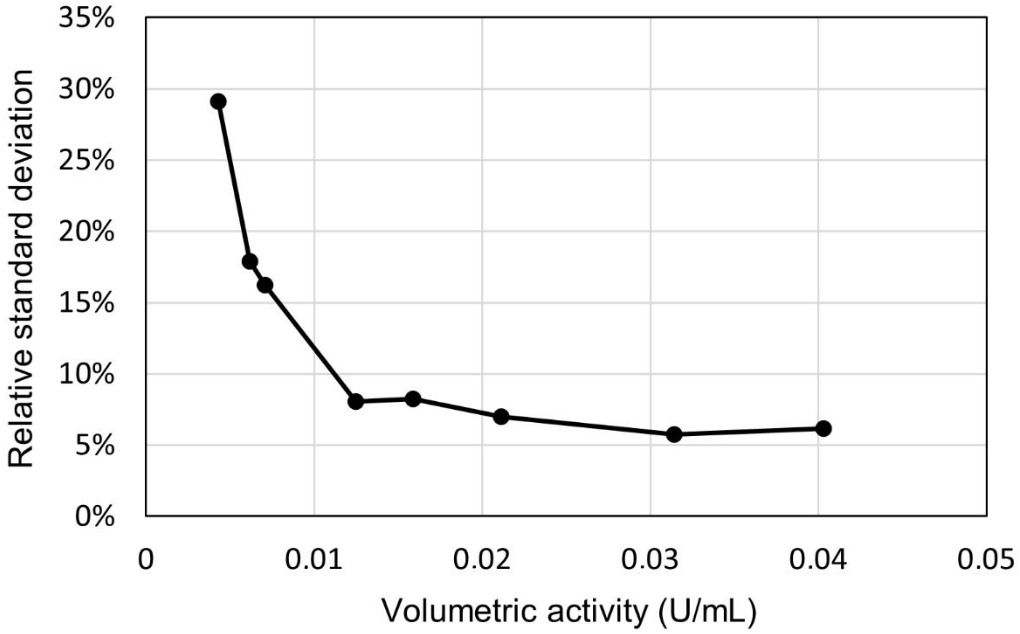

Figure S4: Assay validation. Relative standard deviation based on five independent measurement replicates measured with the cyt *c* assay in a plate reader setup.

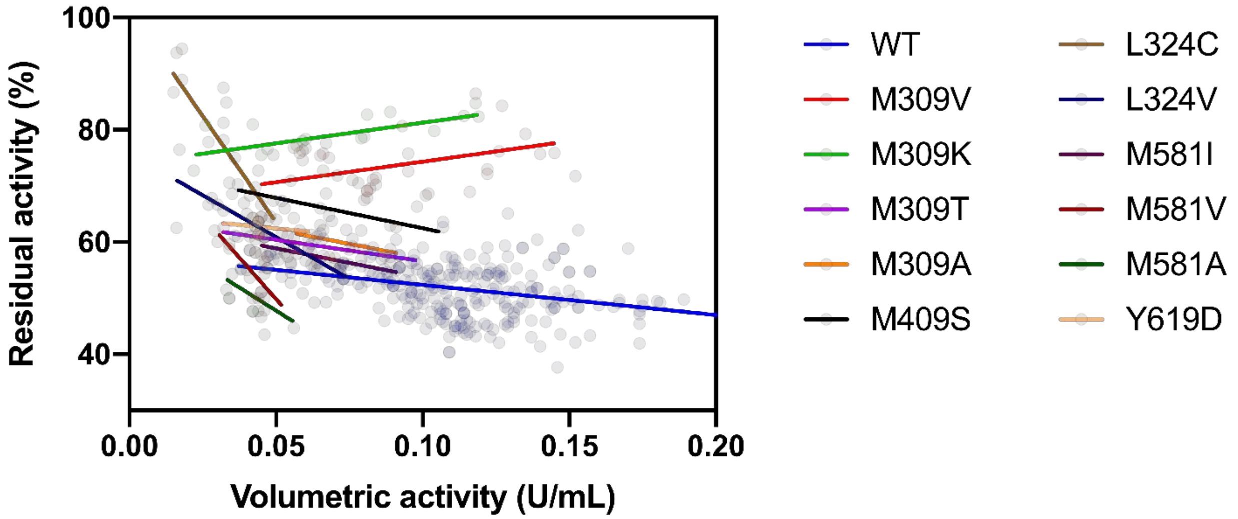

Figure S5: Residual activity of identified hits in dependence of volumetric activity. Linear regression analysis of initial volumetric activity against residual activity was performed to evaluate the relation of activity to residual activity.

Table S1: Summary of results from the screening and rescreening process.

Variant	Screened colonies	Number of hits			% active variants/ colonies[1]	Screening quality (wild-type supernatant)				
		Screening	Rescreening	Variants		% > 0.03 U/mL	Vol. activity (U/mL) MV	Vol. activity (U/mL) SD	Residual activity MV	Residual activity SD
**Active site**										
K299D	8	activity too low (100 % < 0.03 U/mL)				94 %	0.066	0.021	58 %	5 %
E304S	8	0			active	94 %	0.066	0.021	58 %	5 %
E304T	8	0			active	94 %	0.066	0.021	58 %	5 %
E603X	400	6	0	0	42 %	91 %	0.038	0.018	52 %	7 %
P694Y	8	activity too low (100 % < 0.03 U/mL)				94 %	0.066	0.021	58 %	5 %
N700X	400	2	0	0	6 %	99 %	0.088	0.027	57 %	6 %
**Putative oxygen reactive center**										
Oxygen channel combinatorial	5120	11	0	0	3 %	97 %	0.063	0.012	53 %	4 %
NAGL loop combinatorial	2000	3	2	2	2 %	98 %	0.072	0.030	58 %	8 %
L324X	400	4	4		14 %	95 %	0.099	0.019	53 %	6 %
F326X	480	5	0	0	15 %	83 %	0.123	0.026	52 %	5 %
Q597X	400	10	0	0	19 %	100 %	0.039[2]	0.014	60 %	3 %
T599X	400	3	0	0	7 %	100 %	0.098	0.015	52 %	3 %
T599H	8	inactive (i.e., not measurable)				94 %	0.066	0.021	58 %	5 %
Y619X	400	5	1	1	7 %	89 %	0.101	0.027	54 %	7 %
T747A	8	0			active	100 %	0.110	0.039	65 %	5 %
T747H	8	0			active	100 %	0.110	0.039	65 %	5 %
N748X	504	12	0	0	4 %	96 %	0.040[2]	0.020	61 %	4 %
N748C	40	inactive (i.e., not measurable)				93 %	0.053	0.026	60 %	8 %
**Structural stability**										
M75X	400	1	0	0	4 %	100 %	0,110	0,017	46 %	10 %
M309X	400	29	20	4	32 %	95 %	0.098	0.019	57 %	6 %
M409X	400	18	9	1	14 %	91 %	0.107	0.022	46 %	3 %
M513X	480	6	0	0	12 %	91 %	0.076	0.017	45 %	8 %
M581X	640	52[2]	8	3	28 %	94 %	0.117	0.027	53 %	5 %
M690X	400	3	0	0	30 %	94 %	0.109	0.026	70 %	6 %
M690L	8	0			active	94 %	0.066	0.021	58 %	5 %
M690Y	8	0			active	94 %	0.066	0.021	58 %	5 %

M703X	400	10	0	0	34 %	100 %	0.125	0.033	52 %	4 %
**Sum**	**13,736**	**162**	**42**	**11**	**MV[3]**	**95 %**	**0.083**	**0.023**	**56 %**	**5 %**

[1] Calculated for an applied threshold of 0.03 U mL^{-1}. Hence, scarcely active variants were not included. Since the number of variants were not necessarily equal within a library, the percentage of active *colonies* is only for the comparison of libraries. Yet, it was assumed that a low number of threshold-surpassing colonies corresponds to a low number of active variants. [2] Only the best nine variants were further investigated. [3] Mean values of library screening events; not weighted for the respective colony numbers. MV: mean value; SD: standard deviation.

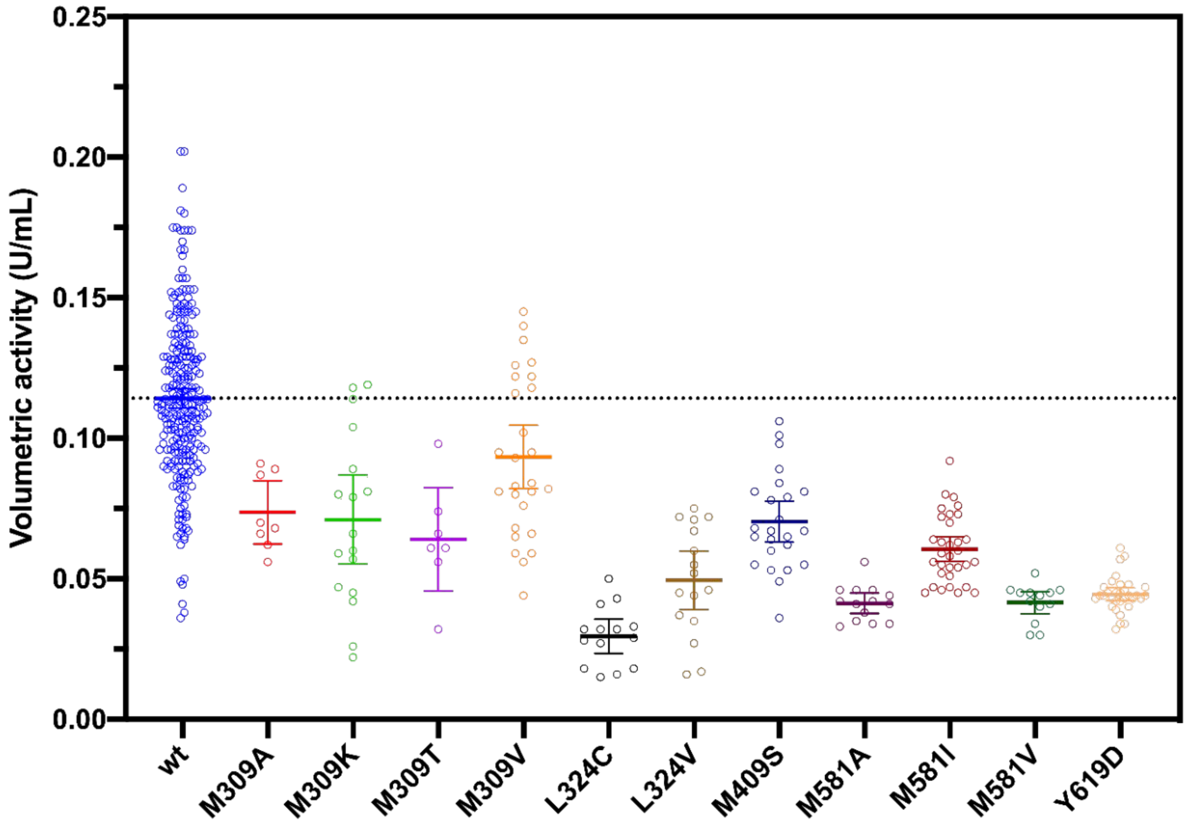

Figure S6: CDH activity in the screening supernatant of identified hits. The volumetric activity is shown as a scatter dot plot with arithmetic means as central line and two error bars at the upper and lower 95 % confidence interval.

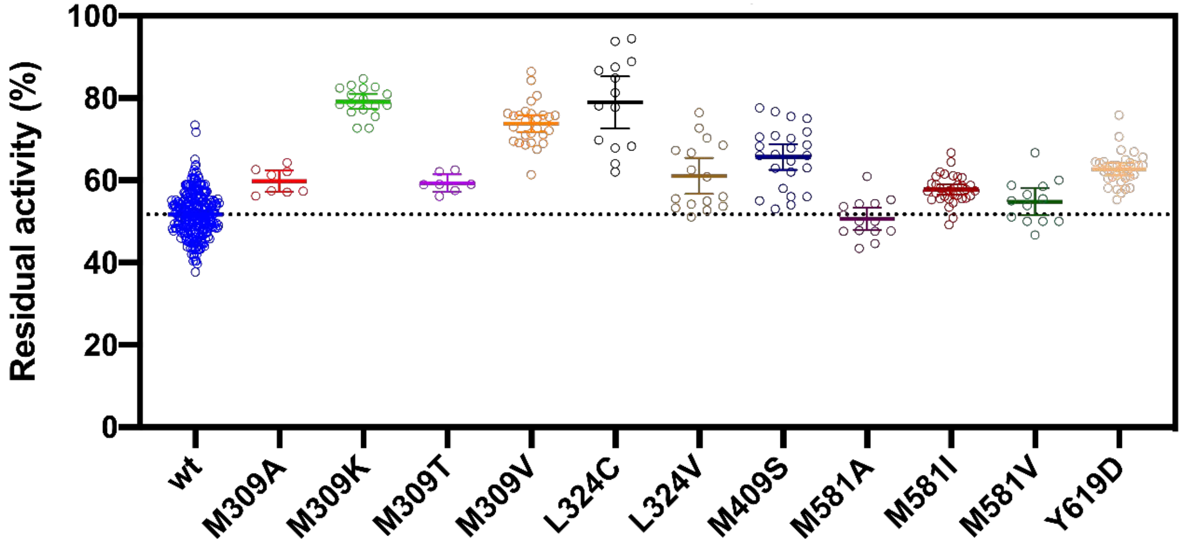

Figure S7: Residual CDH activity of identified variants. Supernatants were incubated for 3 h at 37 °C in 11 mM PBS buffer at pH 7.4 containing 150 mM glucose. The residual CDH activity is shown as percentage regarding the initial activity of the respective variant. Central lines indicate the arithmetic mean. The upper and lower error bars show the 95 % confidence interval.

Table S2: Purification of His-tagged CDH variants by using IMAC. CDH activity was measured by using the standard cytochrome *c* assay at 30 °C with 300 mM glucose as the substrate.

Variants	Total Units	Purification yield [%]	Specific activity [U/mg]	Purity number[1] (A_{420}/A_{280})	Total protein [mg]
Wild-type	248	38	2.44 ± 0.04	0.54 ± 0.03	38.5
M309V	60	41	1.71 ± 0.06	0.57 ± 0.03	17.9
M409S	57	9	0.64 ± 0.02	0.59 ± 0.01	7.8
L324C	38	43	1.06 ± 0.05	0.55 ± 0.03	15.1
M309K/M409S	81	23	0.320 ± 0.001	0.51 ± 0.02	57
M309K/L324C	18	39	0.53 ± 0.01	0.53 ± 0.02	12.9
M309K	86	46	2.07 ± 0.11	0.60 ± 0.05	19
L324C/M409S	43	18	0.64 ± 0.03	0.55 ± 0.02	12.3

[1] The ratio of A_{420}/A_{280} normalizes the amount of heme cofactor by the amount of protein to assess the purity of the prepared enzyme. A purity number greater than 0.5 indicates a purity of >85%.

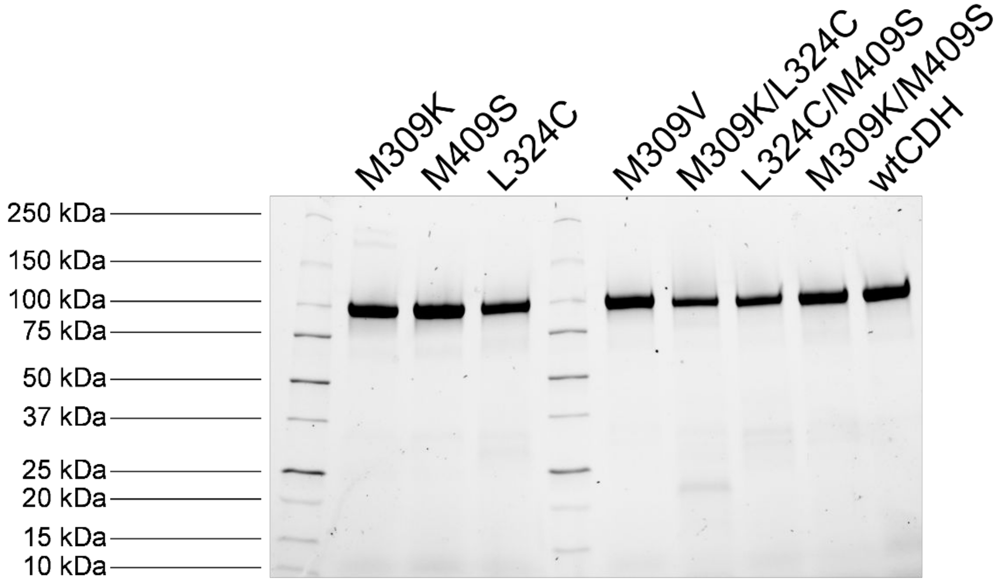

Figure S8: Sodium dodecyl sulfate polyacrylamide gel electrophoresis (SDS-PAGE) of purified CDH variants. 15 µL of the purified CDH variants at their concentrations obtained after purification and rebuffering to 1 mM phosphate buffer containing 0.02 % Triton X-100 (5.6-16.8 mg mL^{-1}) were mixed with 15 µL 2x Laemmli buffer (BioRad) and incubated at 95 °C for 5 min prior to cooling on

ice for 5 min. 25 µL thereof were applied to a stain-free SDS gel (BioRad) as indicated by the legend. 5 µL of Precision Plus Protein™ Standard (BioRad) were applied as standard. The gel ran at 150 V for 55 min. Imaging was done at a BioRad GelDoc station.

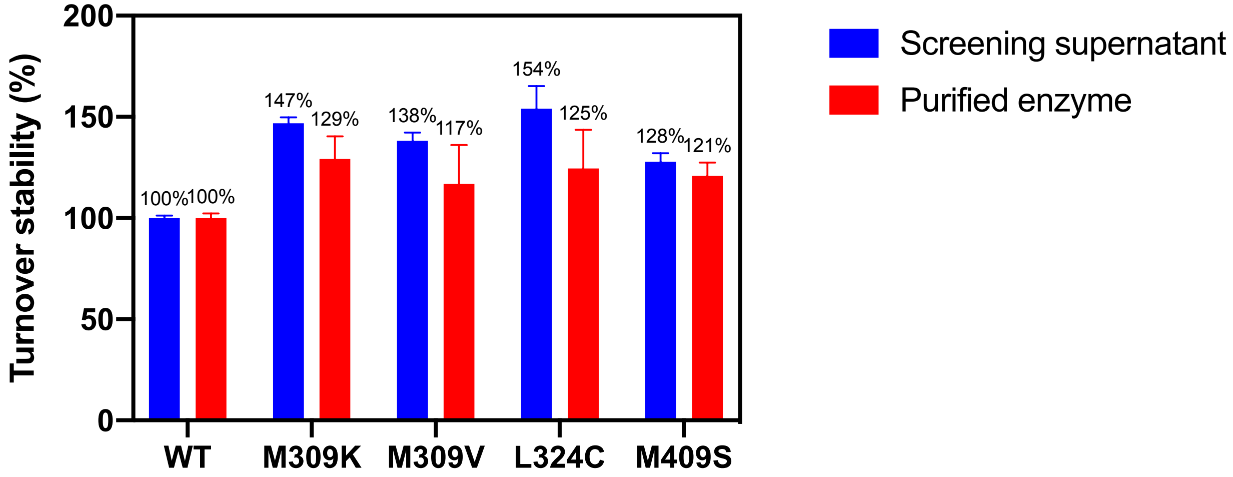

Figure S9: Comparison between the screening results of crude, enzyme-containing supernatants and biochemical characterization of purified enzyme variants. Crude, enzyme-containing supernatants or purified variants (1 mg mL^{-1} in 11 mM buffer, pH 7.4) were incubated at 37 °C in presence of 150 mM glucose and oxygen/air for 3 h. The residual activity expressed as the fraction of the initial activity was normalized to the wild-type activity to obtain the relative turnover stability.

Table S3: Comparison of screening results from crude, enzyme-containing supernatants and the characterization of purified enzymes. The reaction rates at screening conditions (150 mM) were obtained via the cytochrome *c* assay. For comparison, both were normalized to the wild-type results.

Variante	Purified enzyme		Crude, enzyme-containing supernatant from screening
	Reaction rate (s^{-1}) at 150 mM glucose	Reaction rate (s^{-1}) at 150 mM glucose normalized to wild-type	Volumetric activity (U mL^{-1}) at 150 mM glucose normalized to wild-type
Wild-type	4.2	100 %	100 %
M309V	3	71 %	78 %
M309K	3.3	79 %	61 %
M409S	2.2	52 %	61 %

L324C	1.6	38 %	26 %

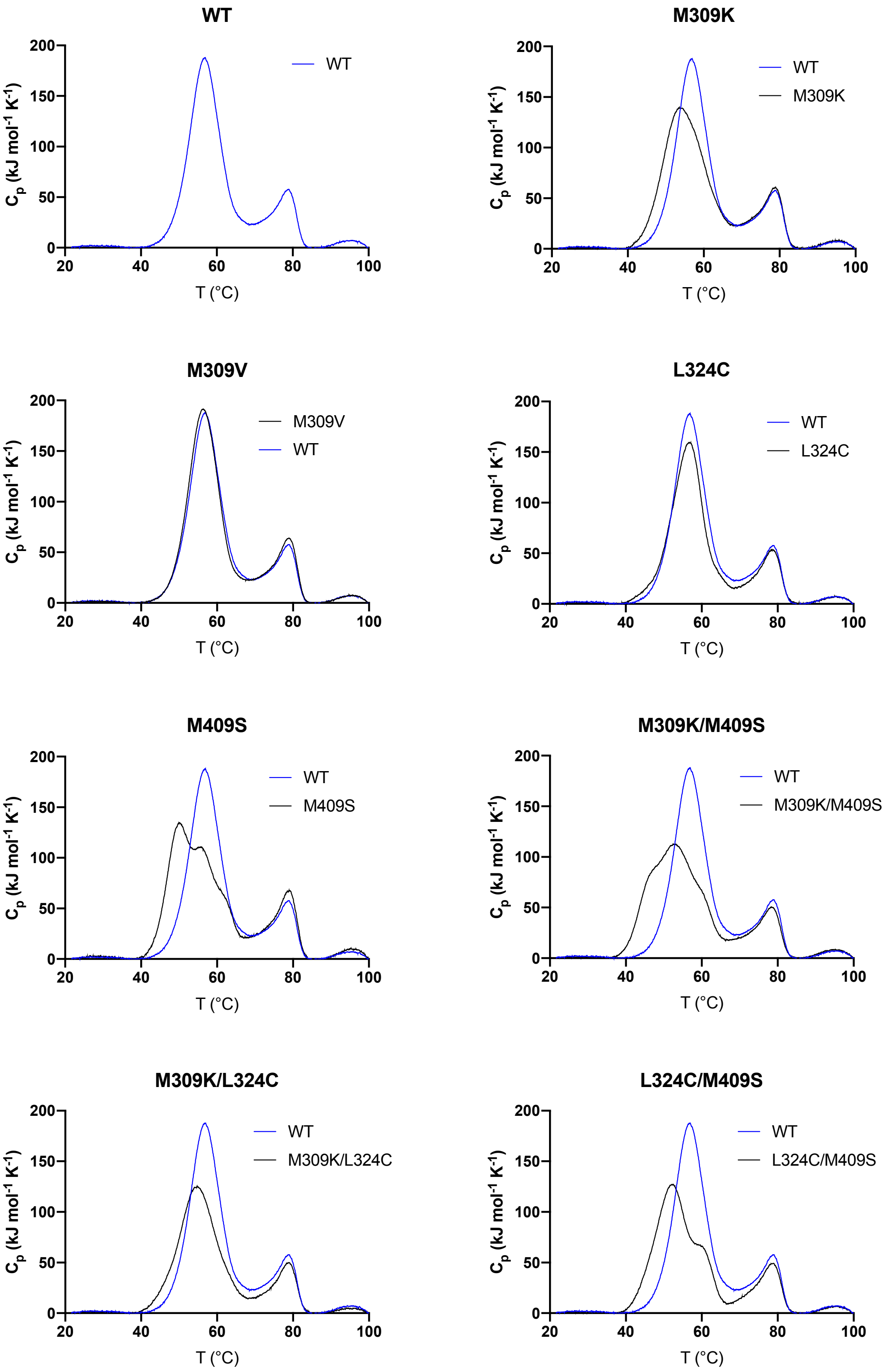

Figure S10: Thermograms of purified CDH variants showing improved turnover stability ('hits'). Differential scanning calorimetry (DSC) of purified variants was performed in 11 mM PBS buffer at pH 7.4 with a protein concentration of 2 mg mL^{-1}. The thermal ramp speed was 1 °C min^{-1}.

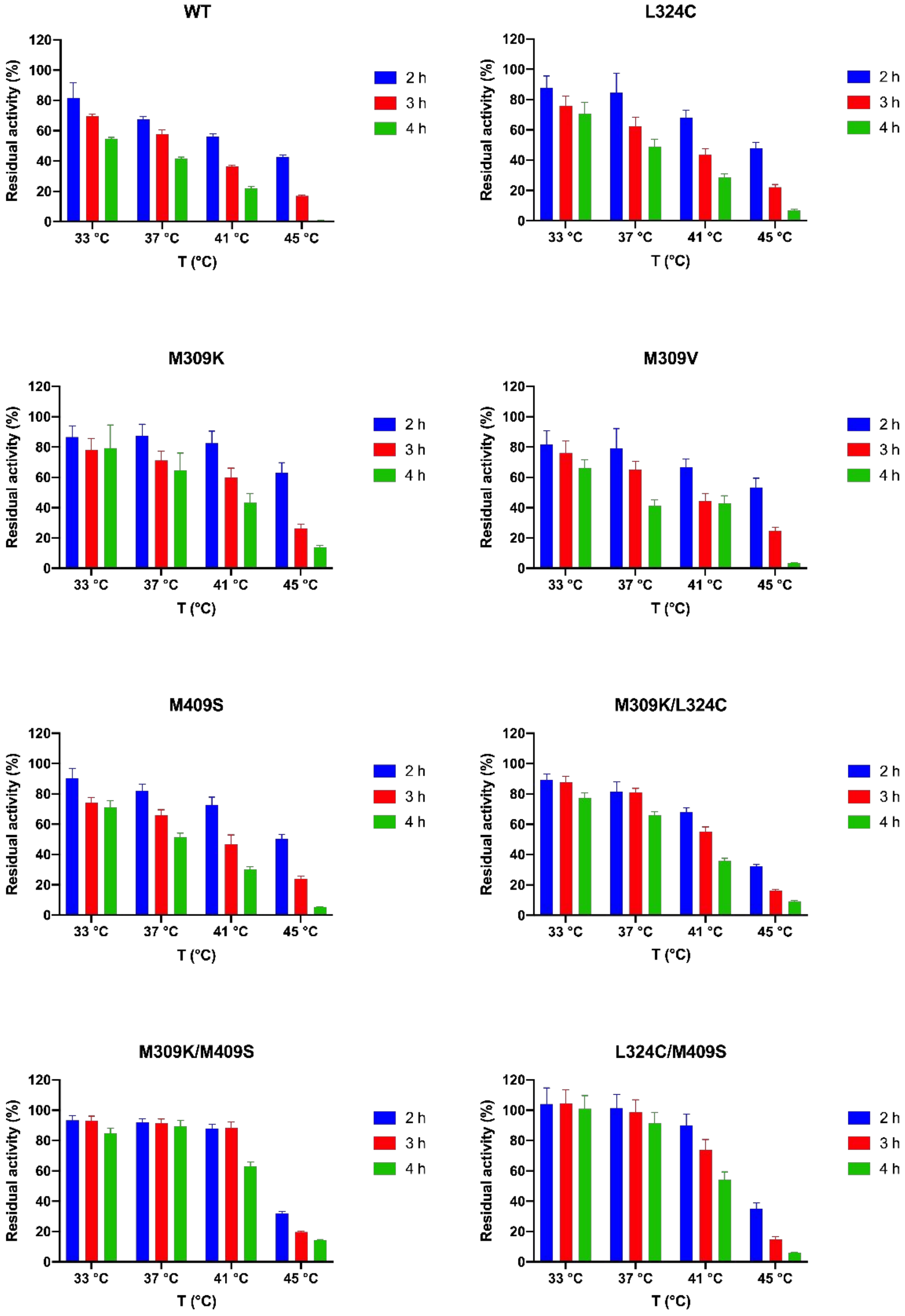

Figure S11: Residual activity of purified CDH variants showing improved turnover stability ('hits'). The purified CDH variants with a final protein concentration of 1 mg mL^{-1} were incubated in 11 mM PBS buffer, pH 7.4, containing 150 mM glucose for 2 h (blue), 3 h (red); and 4 h (green) at the indicated temperatures (33-45 °C) in oxygen/air-saturated buffer. Residual activities were determined with the cytochrome *c* assay and normalized to the initial activity of each variant.

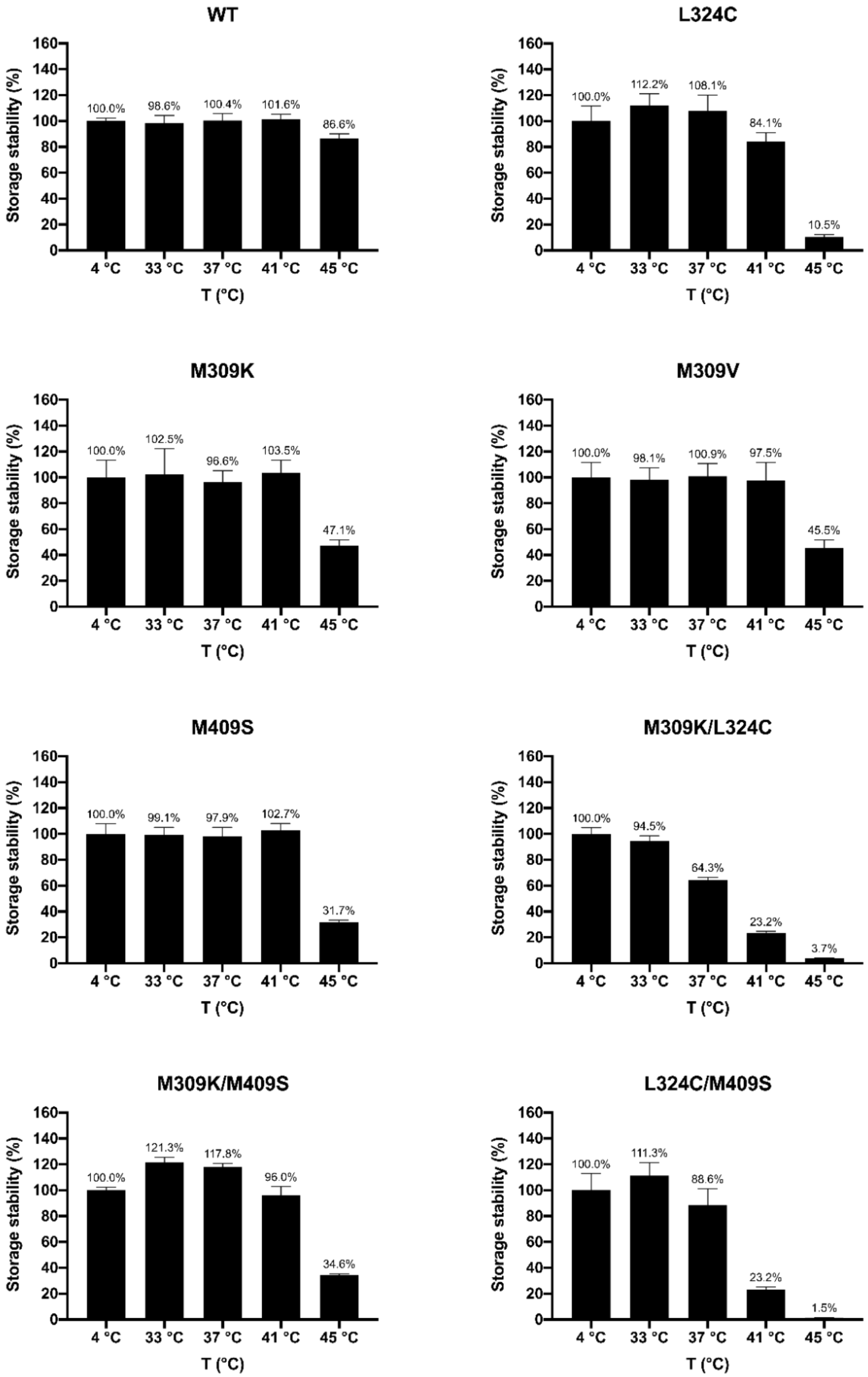

Figure S12: Storage stability of purified CDH variants showing improved turnover stability ('hits'). Purified variants were incubated for 4 h at the indicated temperatures in 11 mM oxygen/air-

saturated PBS buffer, pH 7.4, with a protein concentration of 1 mg mL^{-1}. Residual activities were determined with the cytochrome *c* assay and normalized to the initial activity of each variant.

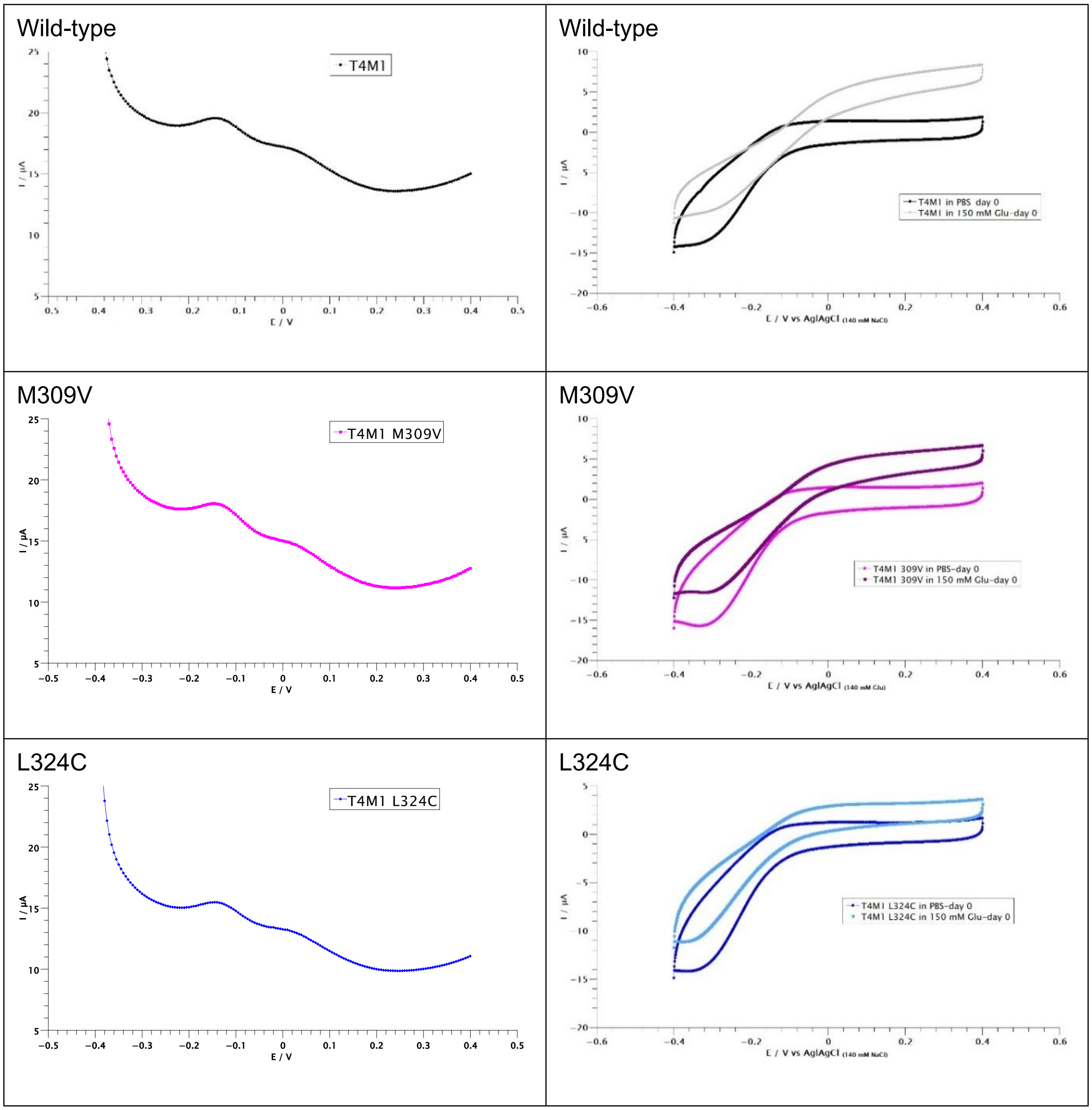

M409S

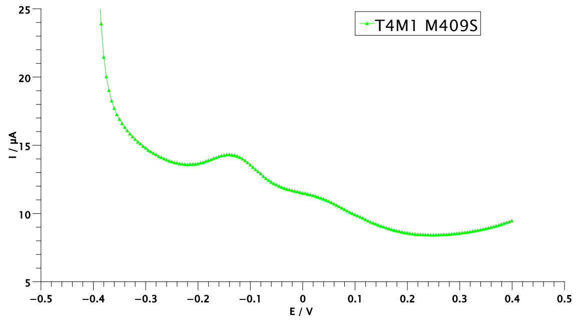

M409S

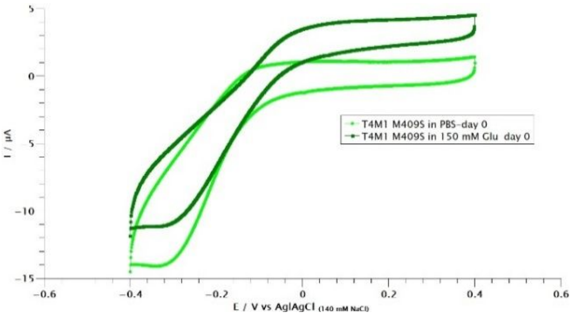

M309K

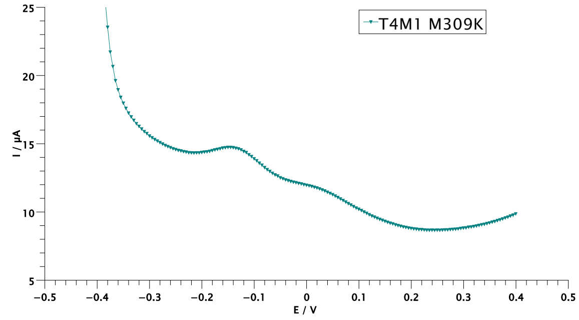

M309K

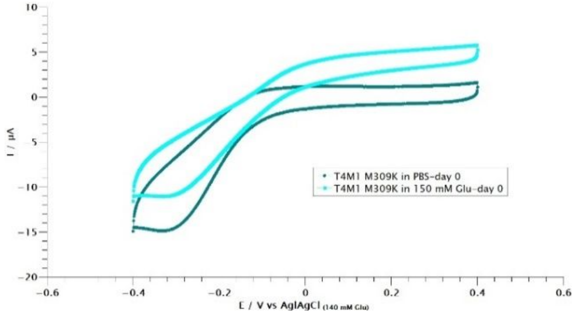

M309K/L324C

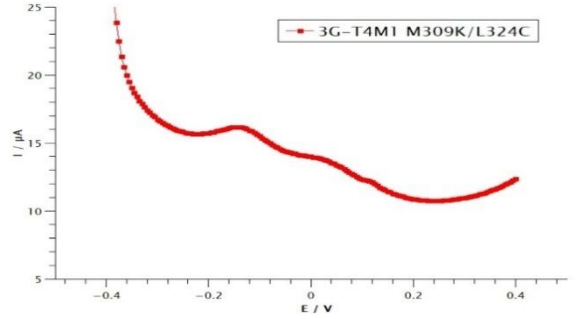

M309K/L324C

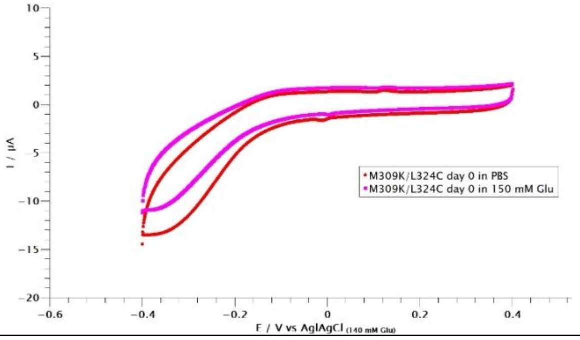

L324C/M409S

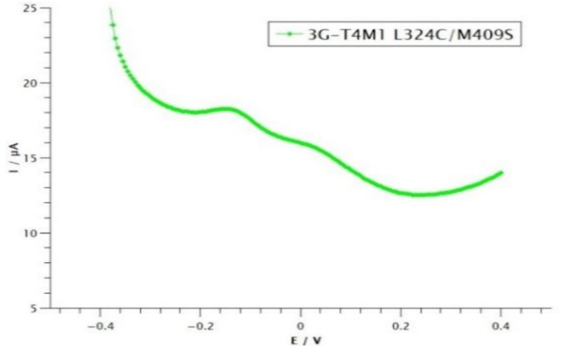

L324C/M409S

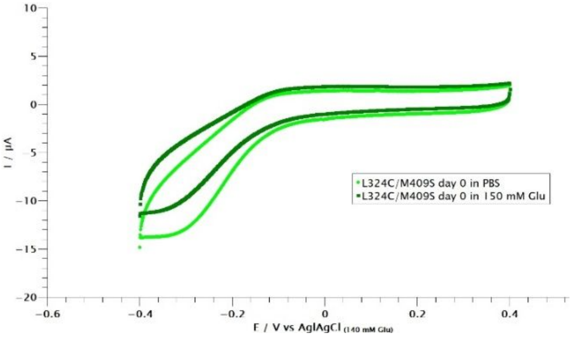

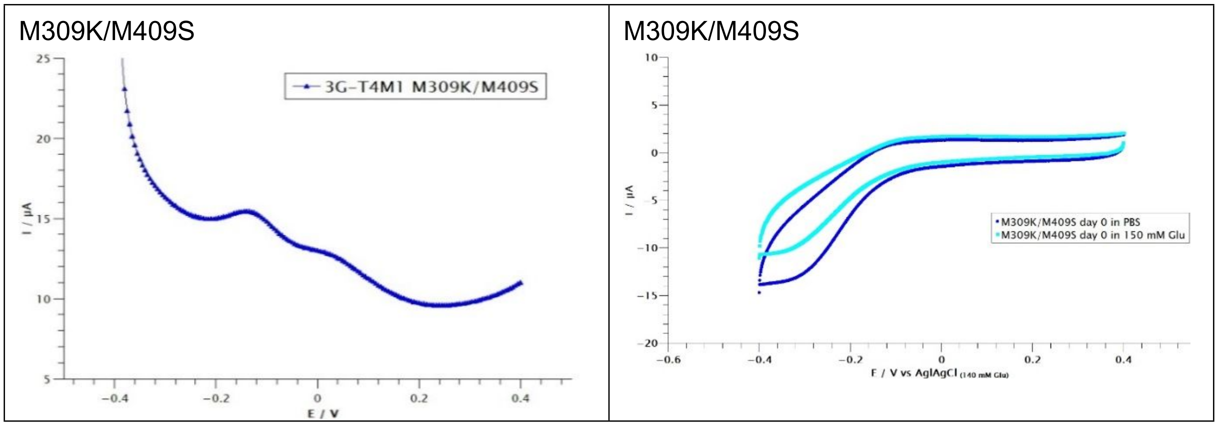

Figure S13: Electrochemical characterization of CDH variants with modified electrodes using square-wave voltammetry in 50 mM potassium phosphate buffer containing 8 g L^{-1} NaCl and 0.2 g L^{-1} KCl, pH 7.4, at 37 °C (left) and cyclic voltammetry measured in the same buffer at 37 °C with and without 150 mM glucose (right).

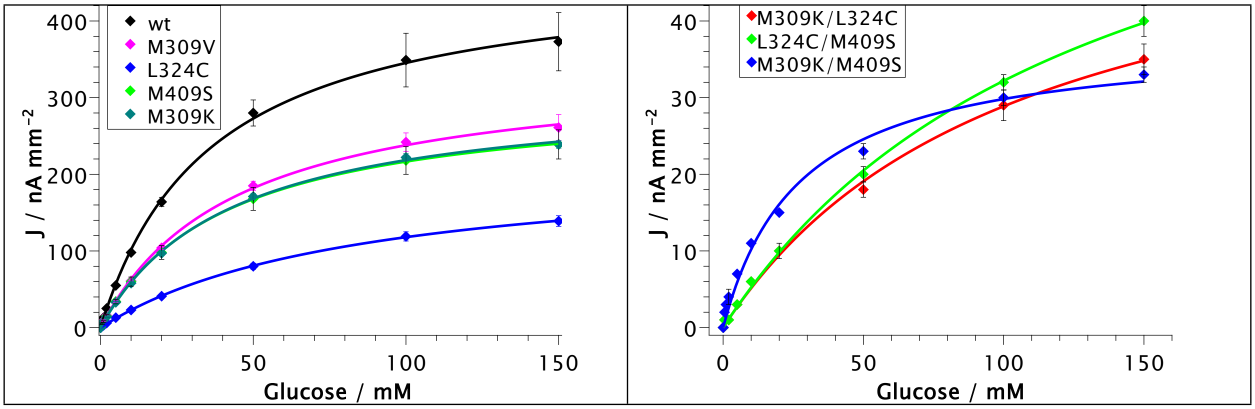

Figure S14: Current densities measured from the amperometric calibration of the CDH variants with various glucose concentrations at 0 V vs. Ag|AgCl in 50 mM potassium phosphate buffer containing 8 g L^{-1} NaCl and 0.2 g L^{-1} KCl, pH 7.4, at 37 °C. The x-axis indicates the glucose concentration in mM while the y-axis indicates the current densities in nA mm^{-2}. Left: single variants, right: double variants.

Table S4: Catalytic parameters derived for glucose as the substrate calculated from fitting amperometric calibration data to the Michaelis-Menten equation.

Variant	K_M (mM)	J_{max} (nA mm^{-2})
Wild-type	36 ± 1	470 ± 6
M309V	44 ± 2	344 ± 5
L324C	86 ± 2	219 ± 3
M409S	41 ± 1	306 ± 2
M309K	42 ± 2	311 ± 4
M309K/L324C	112 ± 5	60 ± 4
L324C/M409S	129 ± 5	75 ± 4
M309K/M409S	28 ± 1	38 ± 1

Table S5. Apparent steady-state catalytic constants derived from steady-state kinetics at 37 °C. Maximal catalytic rate (k_{cat}) and Michaelis-Menten constant (K_M) for glucose when using the electron acceptors DCIP, cytochrome *c*, and Amplex Red at fixed concentrations are shown. The 95 % confidence intervals are shown in parentheses.

Electron acceptor	DCIP		Cytochrome *c*		Amplex Red	
Catalytic constant	k_{cat} (s^{-1})	K_M (mM)	k_{cat} (s^{-1})	K_M (mM)	k_{cat} (s^{-1})	K_M (mM)
**Wild-type**	17.4 (16.3–18.5)	138 (116–161)	6.3 (6.1–6.4)	73 (66–80)	0.105 (0.098–0.112)	81 (64–98)
**M309V**	15.1 (14.1–16.1)	196 (166–226)	4.2 (3.8–4.6)	56 (34–79)	0.122 (0.11–0.133)	130 (97–164)
**M309K**	13 (12.3–13.7)	200 (174–225)	6.2 (6–6.5)	134 (122–147)	0.073 (0.068–0.077)	72 (57–86)
**M409S**	7.1 (6.7–7.4)	84 (71–96)	3 (2.9–3)	48 (43–53)	0.039 (0.036–0.041)	41 (29–52)
**M309K/M409S**	1.6 (1.5–1.7)	44 (36–53)	0.5 (0.5–0.6)	31 (27–35)	0.013 (0.013–0.013)	9 (8–10)
**L324C**	8.6 (7.7–9.5)	205 (156–254)	3.1 (2.9–3.2)	130 (110–150)	0.049 (0.046–0.053)	118 (94–141)
**L324C/M409S**	3.7 (3.3–4.2)	219 (163–274)	1.2 (1.2–1.3)	120 (109–131)	0.0119 (0.0115–0.0123)	11 (9–12)
**M309K/L324C**	3.1 (2.8–3.4)	350 (291–410)	1.2 (1.2–1.3)	245 (223–266)	0.0123 (0.0122–0.0125)	23 (22–24)

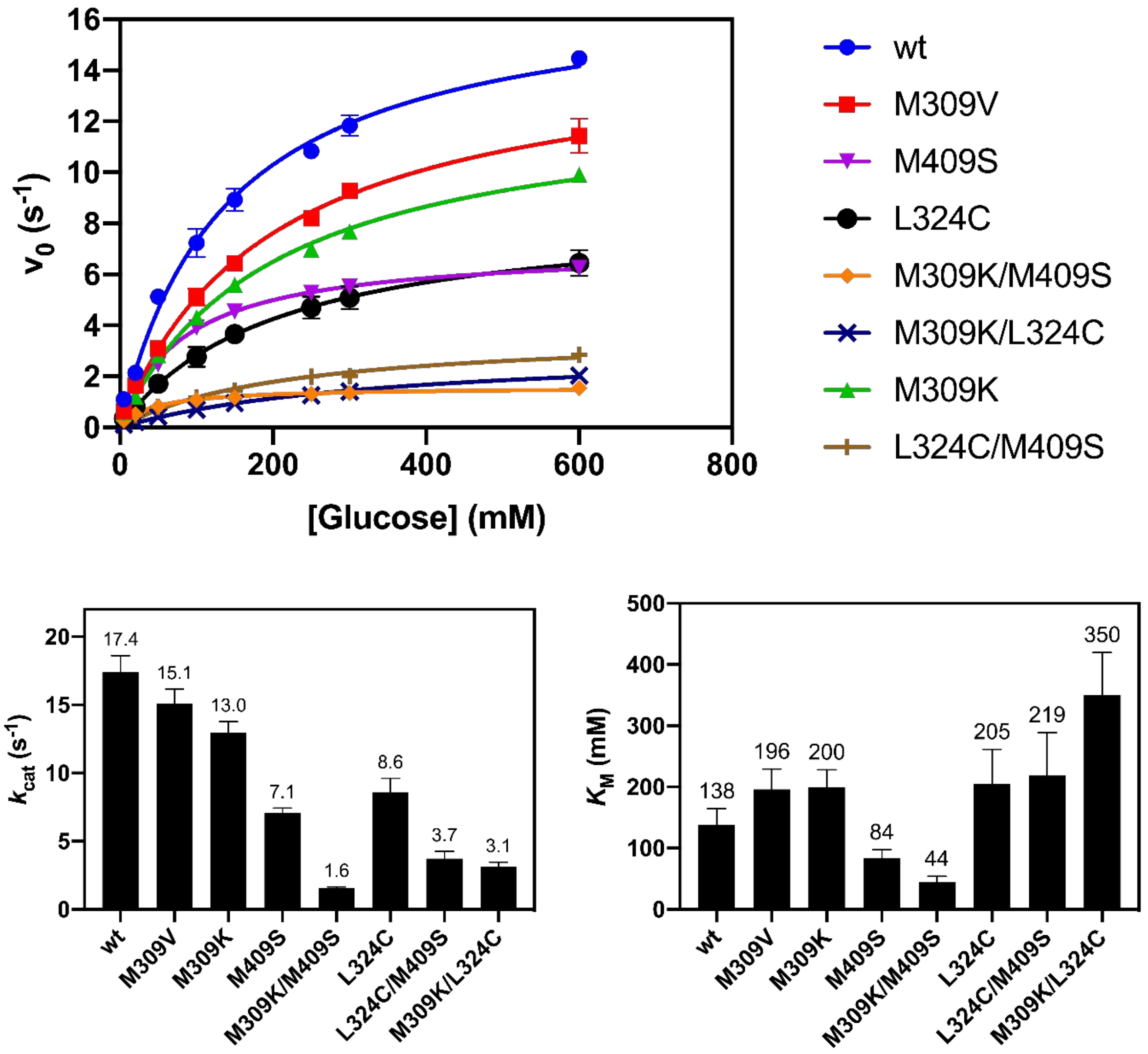

Figure S15: Michaelis-Menten kinetics of purified CDH variants determined with the DCIP assay. The initial velocities v_0 (s^{-1}) are plotted against the glucose concentration in the range of 0–600 mM and fitted using the Michaelis-Menten equation (upper graph). The maximal catalytic rate k_{cat} (bottom graph left) and the Michaelis-Menten constant K_M (bottom graph right) are given as bar charts for each variant. Error bars indicate the standard deviation of two replicates.

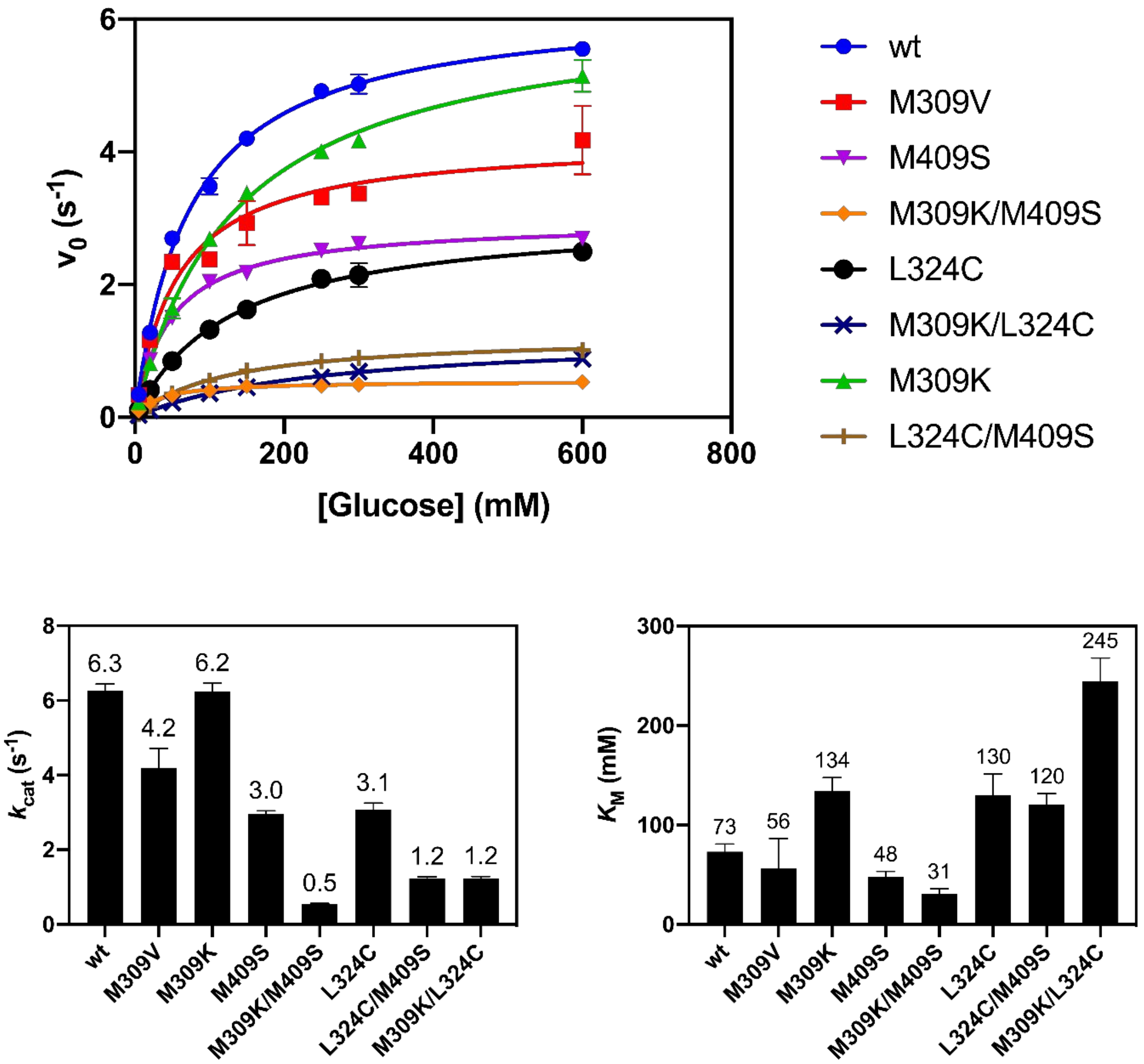

Figure S16: Michaelis-Menten curves of purified CDH variants determined with the cyt *c* assay. The initial velocities v_0 (s^{-1}) of each variant are plotted against the glucose concentration in the range of 0–600 mM using the Michaelis-Menten equation (upper graph). The maximal catalytic rate k_{cat} (bottom left) and Michaelis-Menten constant K_M (bottom right) are shown as bar charts for each variant. Error bars indicate the standard deviation.

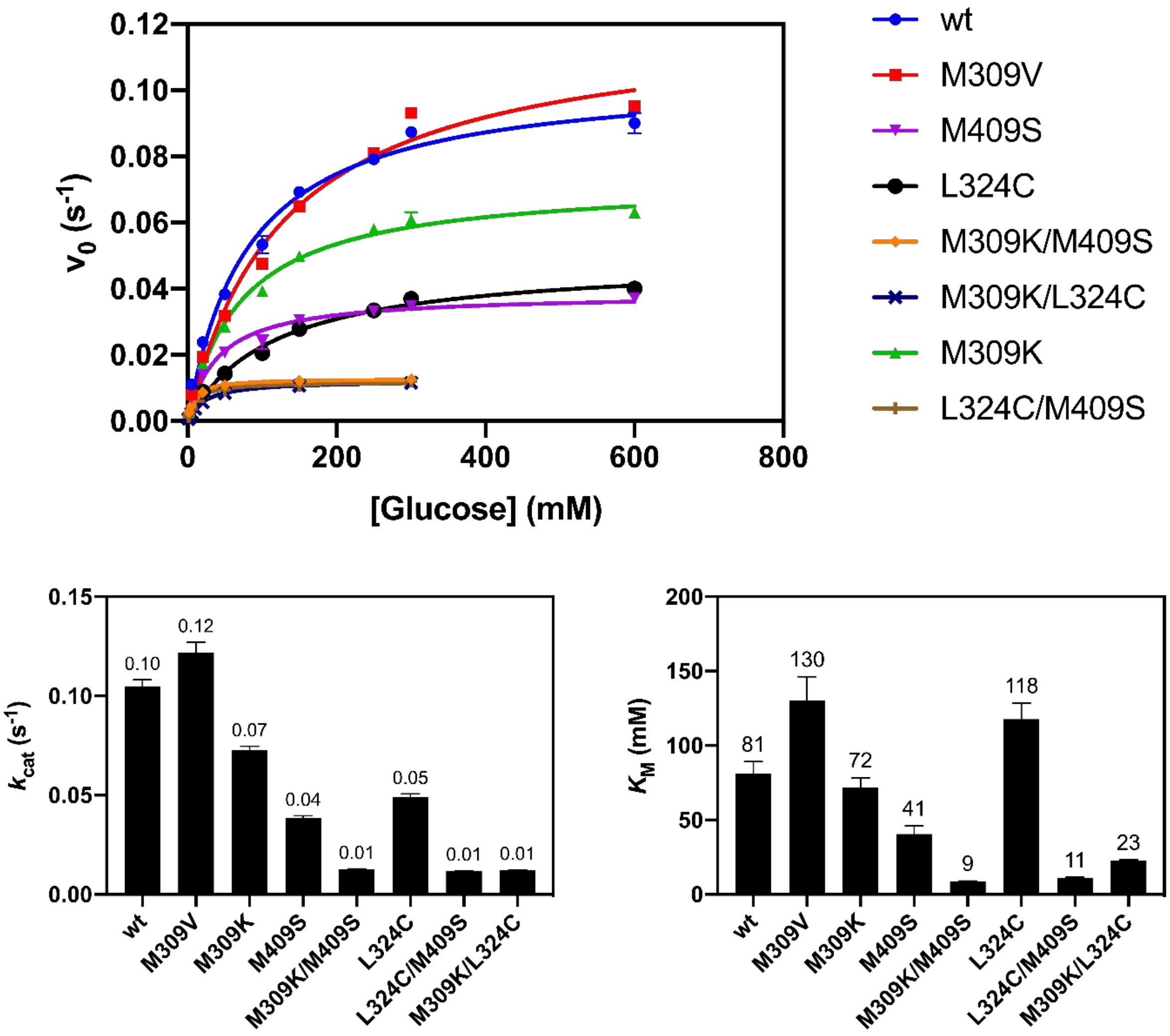

Figure S17: Michaelis-Menten curves of purified hit variants determined with the Amplex Red assay. The initial velocities v_0 (s^{-1}) are plotted against the glucose concentration in the range of 0-600 mM using the Michaelis-Menten equation (upper). The maximal catalytic rate k_{cat} (bottom left) and Michaelis-Menten constant K_M (bottom right) are shown as bar charts for each variant. Error bars indicate the standard deviation.
